# RYBP-PRC1 Complexes Mediate H2A Ubiquitylation at Polycomb Target Sites Independently of PRC2 and H3K27me3

**DOI:** 10.1016/j.cell.2011.12.029

**Published:** 2012-02-17

**Authors:** Lígia Tavares, Emilia Dimitrova, David Oxley, Judith Webster, Raymond Poot, Jeroen Demmers, Karel Bezstarosti, Stephen Taylor, Hiroki Ura, Hiroshi Koide, Anton Wutz, Miguel Vidal, Sarah Elderkin, Neil Brockdorff

**Affiliations:** 1 Department of Biochemistry, University of Oxford, South Parks Road, Oxford OX1 3QU, UK; 2 Nuclear Dynamics, Babraham Institute, Babraham Research Campus, Cambridge CB22 3AT, UK; 3 Mass Spectrometry, Babraham Institute, Babraham Research Campus, Cambridge CB22 3AT, UK; 4 Department of Cell Biology, Erasmus Medical Center, Dr. Molewaterplein 50, 3015GE Rotterdam, The Netherlands; 5 Proteomics Center, Erasmus Medical Center, Dr. Molewaterplein 50, 3015GE Rotterdam, The Netherlands; 6 Computational Biology Research Group, WIMM, University of Oxford, John Radcliffe Hospital, Headington, Oxford OX3 9DS, UK; 7 Department of Stem Cell Biology, Graduate School of Medical Science, Kanazawa University, 13-1 Takaramachi, Kanazawa, Ishikawa 920-8640, Japan; 8 Wellcome Trust Centre for Stem Cell Research, Tennis Court Road, Cambridge CB2 1QR, UK; 9 Cell Proliferation and Development, Centro de Investigaciones Biológicas, Consejo Superior de Investigaciones Cientificas (CSIC), 28040 Madrid, Spain

## Abstract

Polycomb-repressive complex 1 (PRC1) has a central role in the regulation of heritable gene silencing during differentiation and development. PRC1 recruitment is generally attributed to interaction of the chromodomain of the core protein Polycomb with trimethyl histone H3K27 (H3K27me3), catalyzed by a second complex, PRC2. Unexpectedly we find that RING1B, the catalytic subunit of PRC1, and associated monoubiquitylation of histone H2A are targeted to closely overlapping sites in wild-type and PRC2-deficient mouse embryonic stem cells (mESCs), demonstrating an H3K27me3-independent pathway for recruitment of PRC1 activity. We show that this pathway is mediated by RYBP-PRC1, a complex comprising catalytic subunits of PRC1 and the protein RYBP. RYBP-PRC1 is recruited to target loci in mESCs and is also involved in Xist RNA-mediated silencing, the latter suggesting a wider role in Polycomb silencing. We discuss the implications of these findings for understanding recruitment and function of Polycomb repressors.

## Graphical abstract

**Graphical Abstract:**

**Figure:**
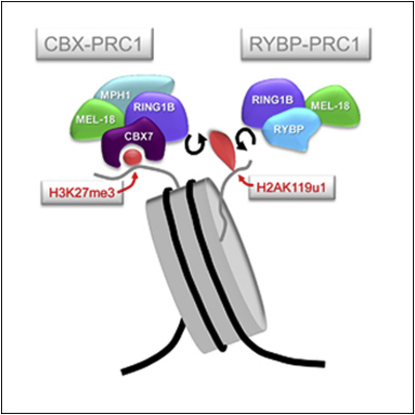

**Highlights:**

► H2A ubiquitylation is retained at Polycomb target loci in the absence of H3K27me3 ► Mutually exclusive complexes, CBX-PRC1 and RYBP-PRC1, mediate H2A ubiquitylation ► RYBP-PRC1 localizes to Polycomb target sites independent of H3K27me3 ► RYBP-PRC1 is required for maintenance of global H2AK119u1 in mESCs

## Teaser

A newly identified variant of the PRC1 complex containing the RYBP subunit allows PRC1 to act independently of PRC2. This alternative complex is important for maintaining the proper chromatin state in ESCs and on the inactive X chromosome.

## Introduction

Polycomb-group (PcG) repressor proteins play a key role in establishing and maintaining gene expression patterns during cellular differentiation and development. There are two major biochemical complexes, PRC1 and PRC2, that have inherent histone-modifying activity critical for their function in gene repression, monoubiquitylation of histone H2AK119 (H2AK119u1), and di- tri-methylation of histone H3K27, respectively (reviewed in Müller and Verrijzer, 2009). Mechanisms other than H2A ubiquitylation also contribute to PRC1-mediated gene repression (Eskeland et al., 2010; Francis et al., 2001, 2004; King et al., 2002; Shao et al., 1999). In mammals the catalytic RING1A/B subunit of PRC1 is also found in the E2F6 (Ogawa et al., 2002; Sánchez et al., 2007; Trimarchi et al., 2001) and BCOR (Gearhart et al., 2006; Sánchez et al., 2007) complexes. An atypical PRC1 complex, dRAF, comprising the proteins dRING, PSC, and the histone demethylase KDM2 has been identified in *Drosophila* (Lagarou et al., 2008).

Genetic analyses have demonstrated that PcG target loci are often coregulated by PRC1 and PRC2, and consistent with this, genome mapping studies in *Drosophila* and mouse demonstrate co-occupancy of PRC1 and PRC2 at many PcG target loci (Boyer et al., 2006; Ku et al., 2008; Schwartz et al., 2006). Co-occupancy is thought to be a consequence of recruitment of PRC1 via interaction of the chromodomain in the PRC1 protein PC (mammalian homologs CBX2/4/6/7/8) with PRC2-dependent H3K27me3. This is based on biochemical studies demonstrating binding of the PC chromodomain to H3K27me3 (Cao et al., 2002; Fischle et al., 2003; Min et al., 2003) and on genetic analyses demonstrating displacement of PRC1 proteins from chromatin in PRC2 mutants (Boyer et al., 2006; Cao et al., 2002; Wang et al., 2004). The idea has been further substantiated in studies demonstrating a direct link between H3K27me3 and PRC1 recruitment (Agger et al., 2007; Lee et al., 2007; Mujtaba et al., 2008).

Although the hierarchical model for PRC1 recruitment is widely accepted, there are specific examples where PRC1/H2AK119u1 targeting is independent of H3K27me3 (reviewed in Simon and Kingston, 2009). Notably, in PRC2-depleted mouse embryonic stem cells (mESCs) (Leeb et al., 2010), and differentiated cells (Pasini et al., 2007), PRC1 proteins have been detected at selected target loci, and moreover, global H2AK119u1 levels are similar to those of wild-type (WT) cells (Schoeftner et al., 2006). Related observations also conflict with hierarchical recruitment. In mESCs, targeting of PRC2 and PRC1 to promoters of key regulators of embryonic lineages is thought to restrain differentiation (Azuara et al., 2006; Boyer et al., 2006; Mikkelsen et al., 2007; Stock et al., 2007). Arguing against this, PRC2-deficient mESCs remain undifferentiated and show only minimal upregulation of PcG target loci (Boyer et al., 2006; Chamberlain et al., 2008; Leeb et al., 2010; Shen et al., 2008). Conversely, PRC1-deficient mESCs strongly upregulate PcG target loci and differentiate spontaneously (Endoh et al., 2008; Stock et al., 2007).

In this study, we investigated PRC1 recruitment in PRC2 null mESCs. We show that in the absence of H3K27me3, PRC1 catalytic subunits occupy the majority of PcG target loci, albeit at reduced levels. This recruitment confers near normal levels of H2AK119u1. We further demonstrate that H3K27me3-independent H2AK119u1 is mediated by a PRC1-related complex, RYBP-PRC1, comprising PRC1 catalytic subunits and the protein RYBP.

## Results

### H2AK119u1 and PRC1 Subunits Localize to PcG Target Genes in *Eed*^−/−^ ESCs

**Figure 1:**
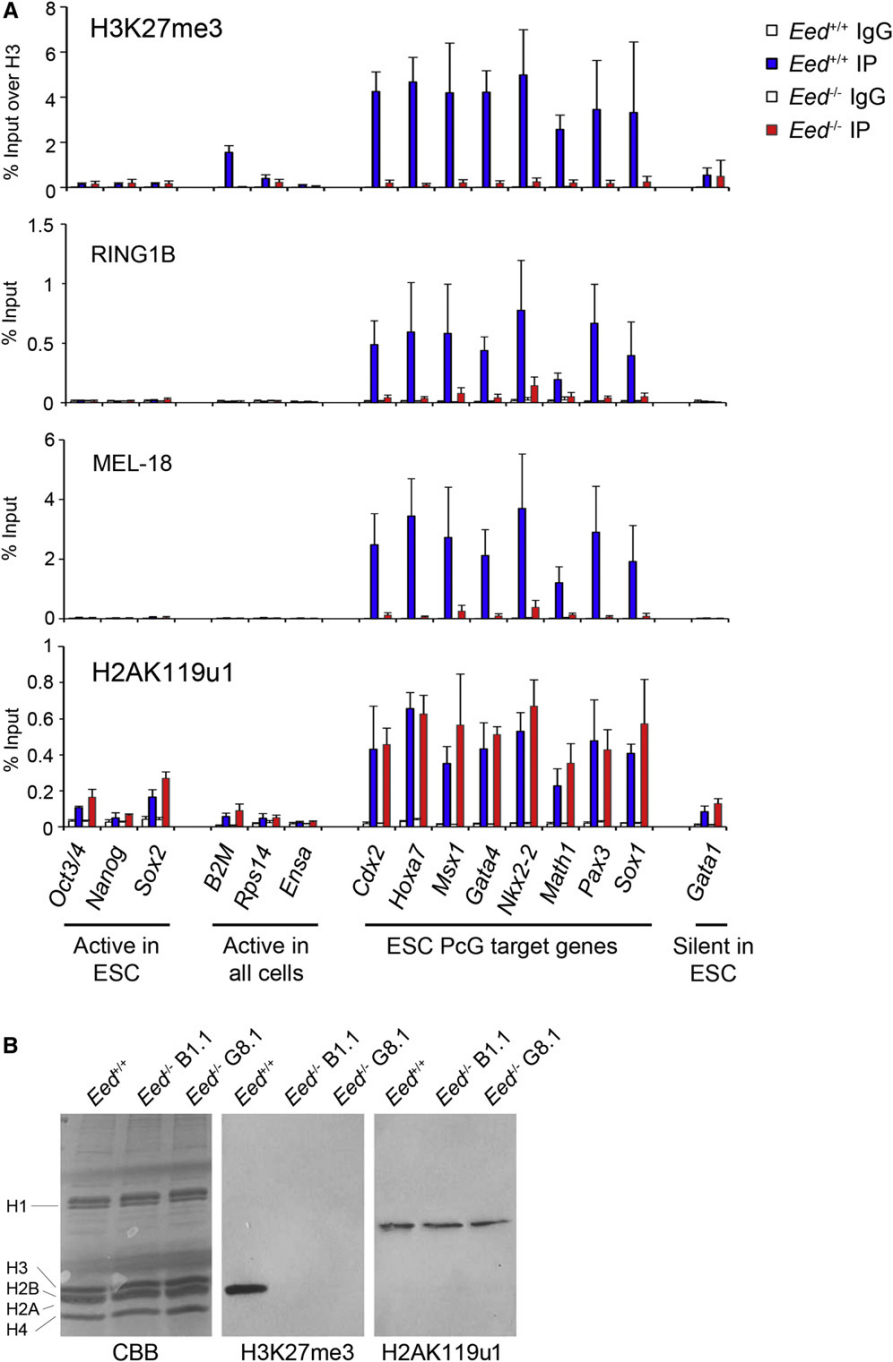
Retention of H2AK119u1 at PcG Target Loci in *Eed^−/−^* mESCs (A) ChIP analysis of H3K27me3, RING1B, MEL-18, and H2AK119u1 in wild-type (*Eed^+/+^*) and *Eed^−/−^* mESCs. Bars show average + SD, n = 3. (B) Western blot analysis of histone extracts showing absence of H3K27me3 and retention of H2AK119u1 in two independent *Eed^−/−^* mESC cell lines, B1.1 and G8.1. CBB (Coomassie brilliant blue). See also Figure S1.

To investigate the importance of H3K27me3 in PRC1 recruitment in mESCs, we performed ChIP for selected PcG target loci in *Eed*^−/−^ mESCs that lack H3K27me3. In addition to loci repressed by PcG proteins, we analyzed loci that are expressed in mESCs, loci that are widely expressed, and a locus that is repressed in mESCs independently of PcG activity. As shown in Figure 1A, H3K27me3 was depleted in *Eed*^−/−^ mESCs, and there was a greatly reduced occupancy of the PRC1 core proteins RING1B and MEL-18 compared to *Eed^+/+^* mESCs. These observations are broadly consistent with hierarchical recruitment of PRC1 by H3K27me3. However, low levels of RING1B/MEL-18 were detectable at PcG target loci in *Eed*^−/−^ cells, and moreover, significant levels of H2AK119u1 were also present (Figure 1A, lower panel). Additionally, global levels of H2AK119u1 were apparently unaffected (Figure 1B).

**Figure S1:**
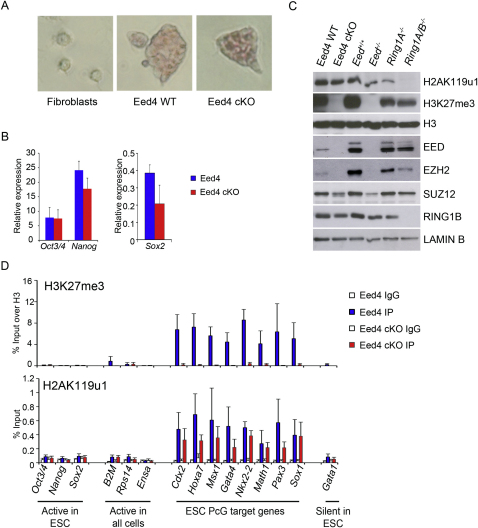
H2AK119u1 Is Retained following Conditional Deletion of *Eed* in mESCs, Related to Figure 1 (A) Alkaline phosphatase staining showing that Eed4 wild-type (WT) and Eed4 cKO cells, 15 days after treatment with 1 μg/ml doxycycline, maintain mESC characteristics. Fibroblasts were used as negative control. (B) Expression analysis of pluripotency genes *Oct3/4*, *Nanog*, and *Sox2* in Eed4 WT and Eed4 cKO 15 days after treatment with 1 μg/ml doxycycline. Expression was normalized against the average of three housekeeping genes, *Hmbs*, *Gapdh*, and *Idh1*. Bars show average + SD, n = 3. (C) Western blot analysis of histone extracts (H2AK119u1, H3K27me3, H3) and nuclear extracts (EED, EZH2, SUZ12, RING1B, and LAMIN B) in wild-type and mutant mESC lines as indicated. (D) ChIP analysis at PcG target and control genes for H3K27me3 and H2AK119ub1 in Eed4 WT and Eed4 cKO mESCs. Bars show average + SD, n = 3.

Similar results were obtained using a conditional knockout (cKO) *Eed*^−/−^ ESC line, Eed4, in which *Eed* is repressed when doxycycline is added to the culture medium (Ura et al., 2008). Treatment of Eed4 cells with doxycycline for 15 days did not affect mESC pluripotency (Figures S1A and S1B available online). Western blot analysis demonstrated that EED protein and H3K27me3 were fully depleted (Figure S1C). Levels of PRC2 core proteins EZH2 and SUZ12 were also strongly reduced (Figure S1C), consistent with previous observations (Pasini et al., 2007). In contrast, global levels of H2AK119u1 were broadly unchanged, as were levels of RING1B (Figure S1C). Analysis of defined PcG target genes by ChIP demonstrated depletion of H3K27me3 and retention of H2AK119u1, albeit at moderately reduced levels (Figure S1D). That lower levels of H2AK119u1 occur at target loci relative to constitutive *Eed*^−/−^ cells (Figure 1A) may indicate that enhanced H2AK119u1 is favored by cell selection during derivation and long-term culture. These results confirm that maintenance of H2AK119u1 in mESCs occurs independently of PRC2 and associated H3K27me3.

### RING1B Is Retained at the Majority of PcG Targets in the Absence of H3K27me3

**Figure 2:**
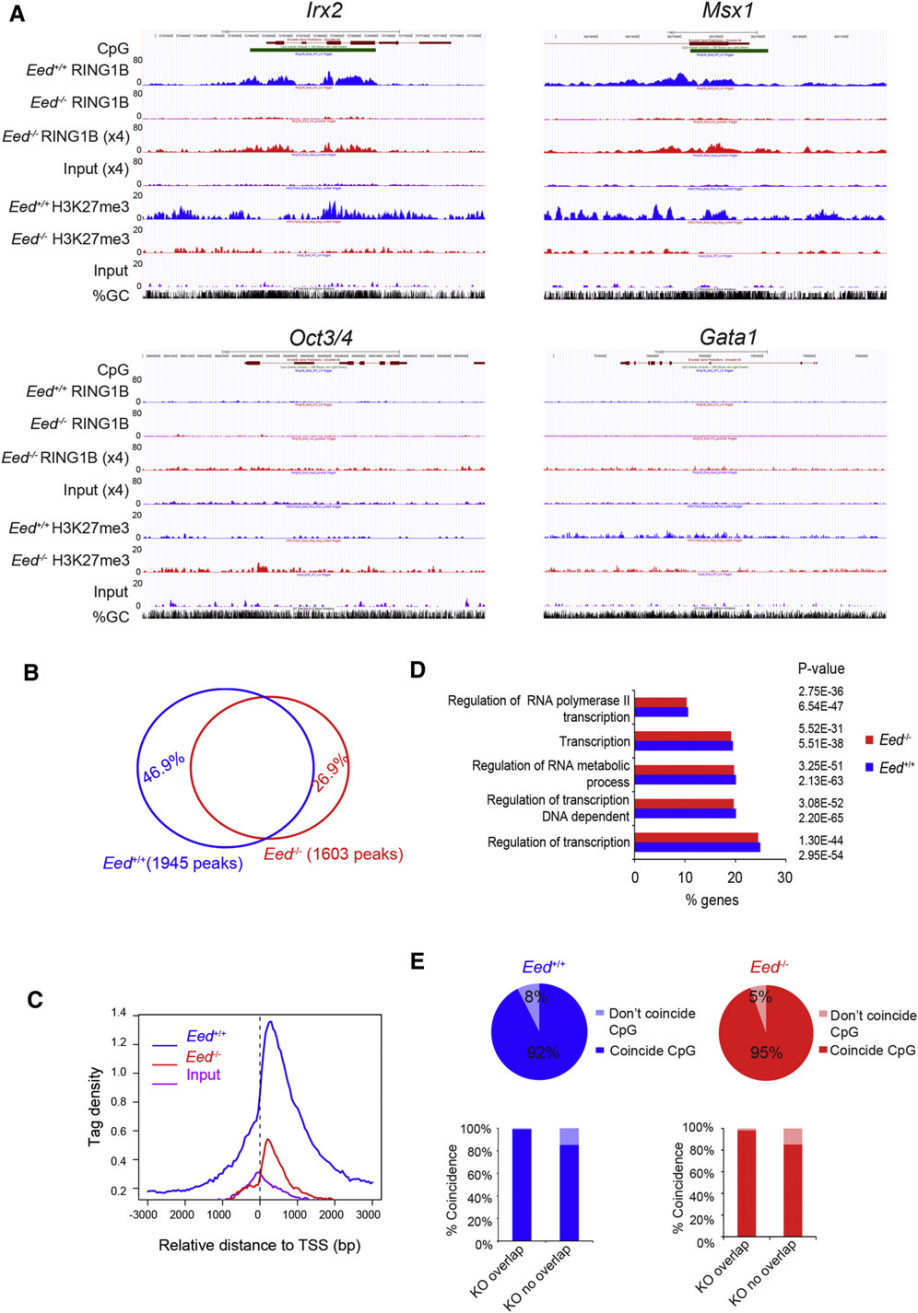
ChIP-Seq Demonstrates RING1B Occupancy at PcG Target Loci in *Eed^+/+^* and *Eed^−/−^* mESCs (A) Example screen shots for *Eed^+/+^* IP (blue) and *Eed^−/−^* IP (red) and input (lilac). *Irx2* and *Msx1* are PcG targets. *Oct3/4* and *Gata1* are non-PcG targets. (B) Venn diagram indicating overlap of RING1B peaks (genes) in *Eed^+/+^*and *Eed^−/−^* cells. (C) Tag density in relation to TSS of 20 million reads randomly subsampled. (D) Comparison showing similarity in gene ontology distribution for peaks in *Eed^+/+^* and *Eed^−/−^* mESCs. (E) Co-incidence of peaks with CpG islands for *Eed^+/+^* and *Eed^−/−^* mESCs (above) and the proportion of peaks coinciding with CpG islands in overlapping and nonoverlapping subgroups defined in (B). See also Figure S2.

**Figure S2:**
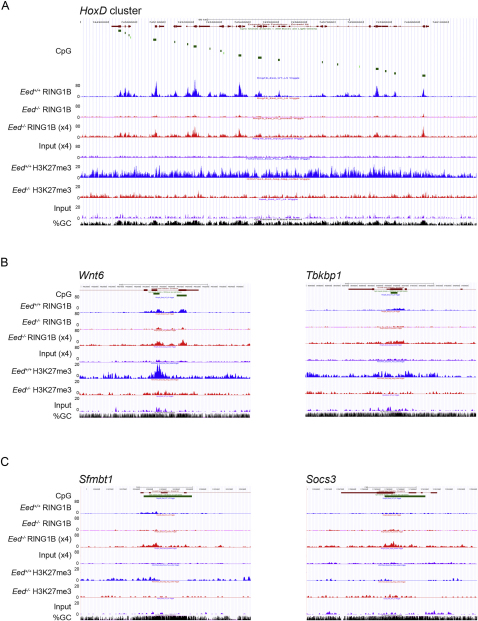
RING1B Occupancy Is Similar in *Eed^+/+^* and *Eed^−/−^* mESCs, Related to Figure 2 Example screen shots illustrate (A) *HoxD*; (B) *Wnt6* and *Tbkbp1* loci, both of which were recorded as peaks in *Eed^+/+^* but not *Eed^−/−^* datasets; and (C) *Sfmbt1* and *Socs3* loci, both of which were recorded as peaks in *Eed^−/−^* but not *Eed^+/+^*datasets. Panels show gene structure, CpG islands, and % GC tracks in addition to distribution of RING1B ChIP-seq reads in *Eed^+/+^* IP (blue) and *Eed^−/−^* IP (red) and input (lilac).

Conventional ChIP analysis indicated that low levels of the core PRC1 proteins RING1B and MEL-18 are present at selected PcG targets following depletion of H3K27me3 in mESCs (Figure 1A). To extend this we carried out RING1B ChIP-sequencing (ChIP-seq) in *Eed^+/+^* and *Eed*^−/−^ ESCs. H3K27me3 ChIP-seq was carried out as a control. *Irx2, Msx1* (Figure 2A), and *HoxD* (Figure S2A) are examples of defined PcG targets in mESCs (Mikkelsen et al., 2007). RING1B binding is readily detectable in *Eed^+/+^* cells and also in *Eed*^−/−^ cells, albeit at much lower levels. To compensate for the reduced signal, we increased the number of reads for the *Eed*^−/−^ RING1B and input samples by approximately 4-fold (Figures 2A and S2A, x4 tracks). This revealed that the broad pattern of RING1B occupancy, mapping to target loci-associated CpG islands, is retained in *Eed*^−/−^ mESCs. Non-PcG target loci, for example *Oct3/4*, a gene that is expressed in ESCs, and *Gata1*, a gene that is silenced independently of PcG, do not show RING1B occupancy either in *Eed^+/+^* or *Eed*^−/−^ ESCs (Figure 2A).

Using model-based analysis of ChIP-Seq (MACS) to identify peaks, we found 2,347 places where RING1B is enriched in *Eed^+/+^* cells, and in comparison 1,810 places in *Eed*^−/−^ cells. Fifty-three percent of regions bound by RING1B in the *Eed^+/+^* ESCs are also targets in the mutant cells (Figure 2B). We consider this to be an underestimate as the thresholds used fail to detect some RING1B peaks over background in *Eed*^−/−^ cells. For example, at the 3′ end of the *Wnt6* locus, MACS records a RING1B peak only for *Eed^+/+^* ESCs, but a similar peak pattern centered on associated CpG islands is seen also in *Eed*^−/−^ ESCs (Figure S2B, left panel). In other cases, for example the *Tbkbp1* locus (Figure S2B, right panel), RING1B peaks recorded in *Eed^+/+^* ESCs are broad and detected only at a low level. In *Eed*^−/−^, this pattern can be observed when comparing to input sample but is not recognized as a peak due to increased background relative to signal. Examples of peaks detected in *Eed*^−/−^ but not *Eed^+/+^* cells are shown in Figure S2C. In some cases, for example *Sfmbt1*, RING1B occupancy is similar in *Eed^+/+^* and *Eed*^−/−^ samples, whereas in others, for example *Socs3*, only very low levels of RING1B occupancy are apparent in the *Eed^+/+^* samples. It is probable that in these examples H3K27me3 is less important for RING1B binding.

The distribution of peaks across transcription start sites (TSS) is similar in *Eed^+/+^* and *Eed*^−/−^, in both cases being within ± 1 Kb from the TSS (Figure 2C). Gene ontology (GO) analysis demonstrates no significant differences between target loci in *Eed^+/+^* and *Eed*^−/−^ samples (Figure 2D).

As noted previously for *Eed^+/+^* cells (Ku et al., 2008) and above (Figures 2 and S2), many of the PcG target regions are CpG islands, with 99% of peaks shared between *Eed^+/+^* and *Eed*^−/−^ cells overlapping with CpG islands (Figure 2E). The few exceptions that we found (13 peaks) are regions with high GC content that are not annotated as CpG islands. Interestingly, a high proportion of the nonoverlapping peaks also coincide with CpG islands (Figures 2E and S2C). Co-occurrence of RING1B sites and CpG was shown to be significant (p < 0.001, co-occurrence of R test; Huen and Russell, 2010). Taken together these results demonstrate extensive overlap of RINGB targets in mESCs lacking H3K27me3 relative to WT cells.

### Re-recruitment of PRC1 Activity to PcG Target Loci following Depletion of H2AK119u1

**Figure 3:**
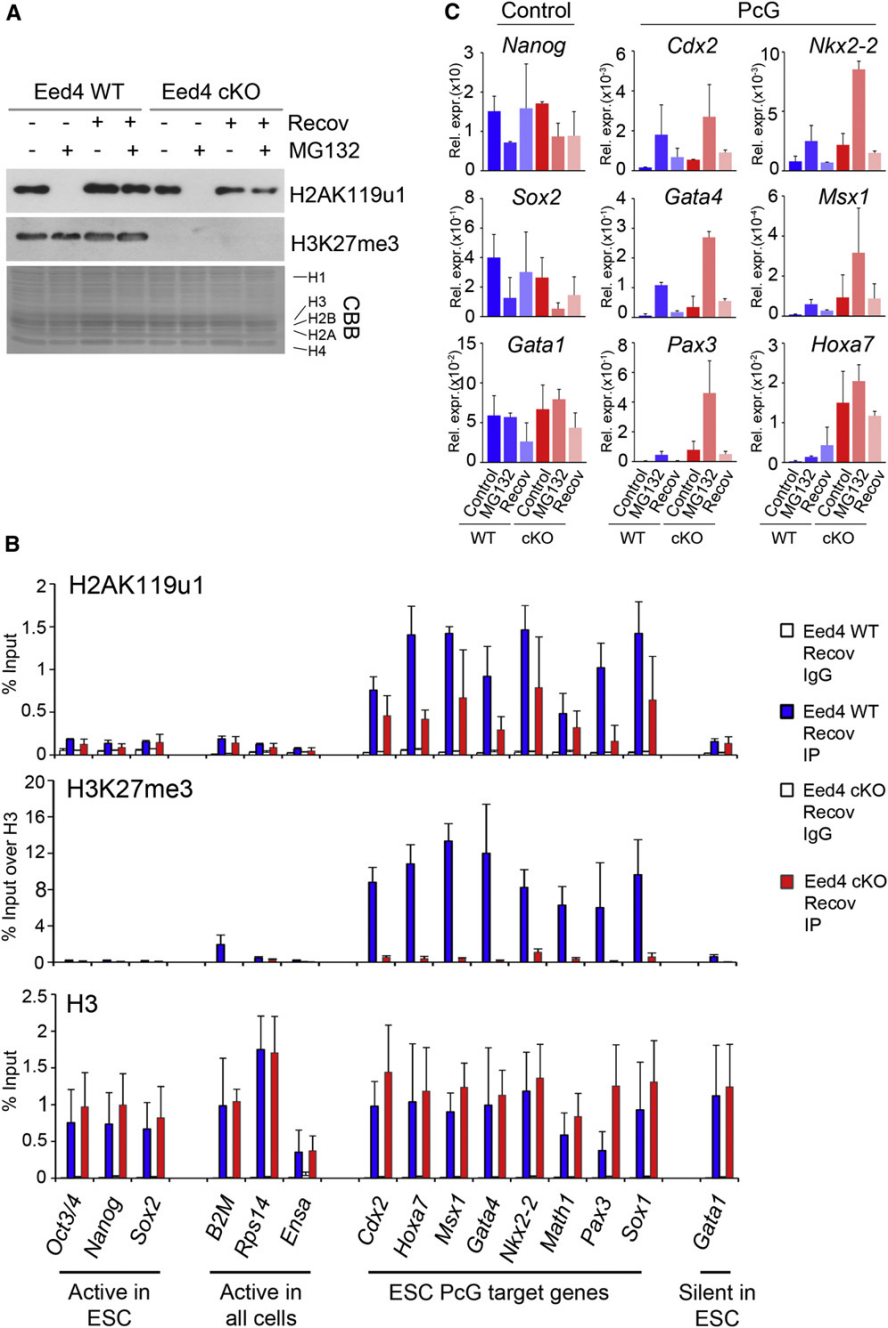
H2AK119u1 Is Re-established following Depletion in Eed4 WT and Eed4 cKO mESCs (A) Western blot for H2AK119u1 and H3K27me3 in histone extracts. CBB: Coomassie brilliant blue. H2AK119u1 is completely depleted after 6 hr with 10 μM MG132 and is then restored when cells are left to recover (recov) for 3 days after inhibitor removal, irrespective of presence of H3K27me3. (B) ChIP for H2AK119u1 and H3K27me3 in Eed4 WT and Eed4 cKO cells. H3 is shown as a control. Bars show average + SD, n = 3. (C) Expression analysis (Rel. expr.) of selected loci. For RT-PCR analysis, values were normalized against the average of three housekeeping genes, *Hmbs*, *Gapdh*, and *Idh1.* Bars show average + SD, n = 3. See also Figure S3.

**Figure S3:**
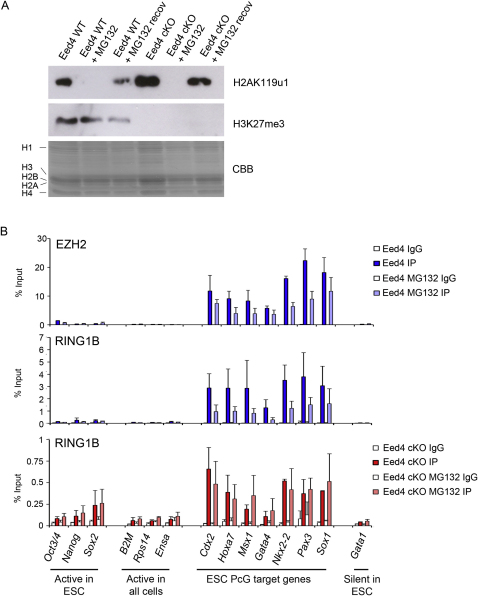
H2AK119u1 Levels Are Re-established 24 hr after Removal of MG132 Inhibitor from the Culture Medium, Related to Figure 3 (A) Western blot of histone extracts shows H2AK119u1 levels before MG132 treatment, during treatment (+MG132) and 24 hr after removal of MG132 (+MG132 recov), in both Eed4 WT and Eed4 cKO ESCs. (B) ChIP analysis of EZH2 in Eed4 WT and RING1B in Eed4 WT and Eed4 cKO mESCs treated with 10 μM MG132 for 6 hr.

PRC2 occupancy and H3K27me3 can be maintained at an ectopic site following withdrawal of the primary recruitment signal (Hansen et al., 2008), possibly via binding of an aromatic cage in EED to H3K27me3 (Margueron et al., 2009). With this in mind, we considered that although primary recruitment of PRC1 may require PRC2-mediated H3K27me3, H2AK119u1, once established, could function as a signal for maintenance of PRC1 occupancy. To address this, we used the reversible proteasome inhibitor MG132 (Dantuma et al., 2006) to deplete H2AK119u1 in Eed4 cells in the presence or absence of PRC2 and then determined whether restoration of H2AK119u1 occurs following withdrawal of the inhibitor. Secondary effects of MG132 treatment on mESCs are negligible with the described conditions (Szutorisz et al., 2006). As shown in Figure 3A, treatment of cells with MG132 for 6 hr efficiently depleted global H2AK119u1 (lanes 2 and 6). Allowing cells to recover for 3 days after withdrawal of the inhibitor resulted in restoration of H2AK119u1 in both the presence and the absence of H3K27me3 (lanes 4 and 8, respectively), and this was also the case after only 1 day of recovery (Figure S3A). ChIP analysis demonstrated that H2AK119u1 accumulates appropriately at PcG target loci after recovery, with levels being slightly reduced in the absence of H3K27me3 (Figure 3B). Recruitment of RING1B and EZH2 was retained following MG132 treatment, albeit at a slightly reduced level (Figure S3B).

Expression analysis (Figure 3C) demonstrated that treatment with MG132 derepresses PcG target loci in both the presence and the absence of H3K27me3, and that silencing is restored following withdrawal of the inhibitor. No effect was seen at the *Gata1* locus at which repression is PcG independent. Initiation of differentiation due to H2AK119u1 depletion (Endoh et al., 2008; Stock et al., 2007) accounts for the reduced levels of expression of pluripotency factors following MG132 treatment. Derepression of PcG targets was enhanced in the absence of H3K27me3, consistent with lower H2AK119u1 when MG132 treatment was begun (see Figure S1C). Taken together these results demonstrate that de novo deposition of H2AK119u1 in mESCs occurs appropriately at known PcG target loci in both the presence and the absence of H3K27me3.

### RYBP Is a Stoichiometric Component of PRC1 in ESCs

**Figure 4:**
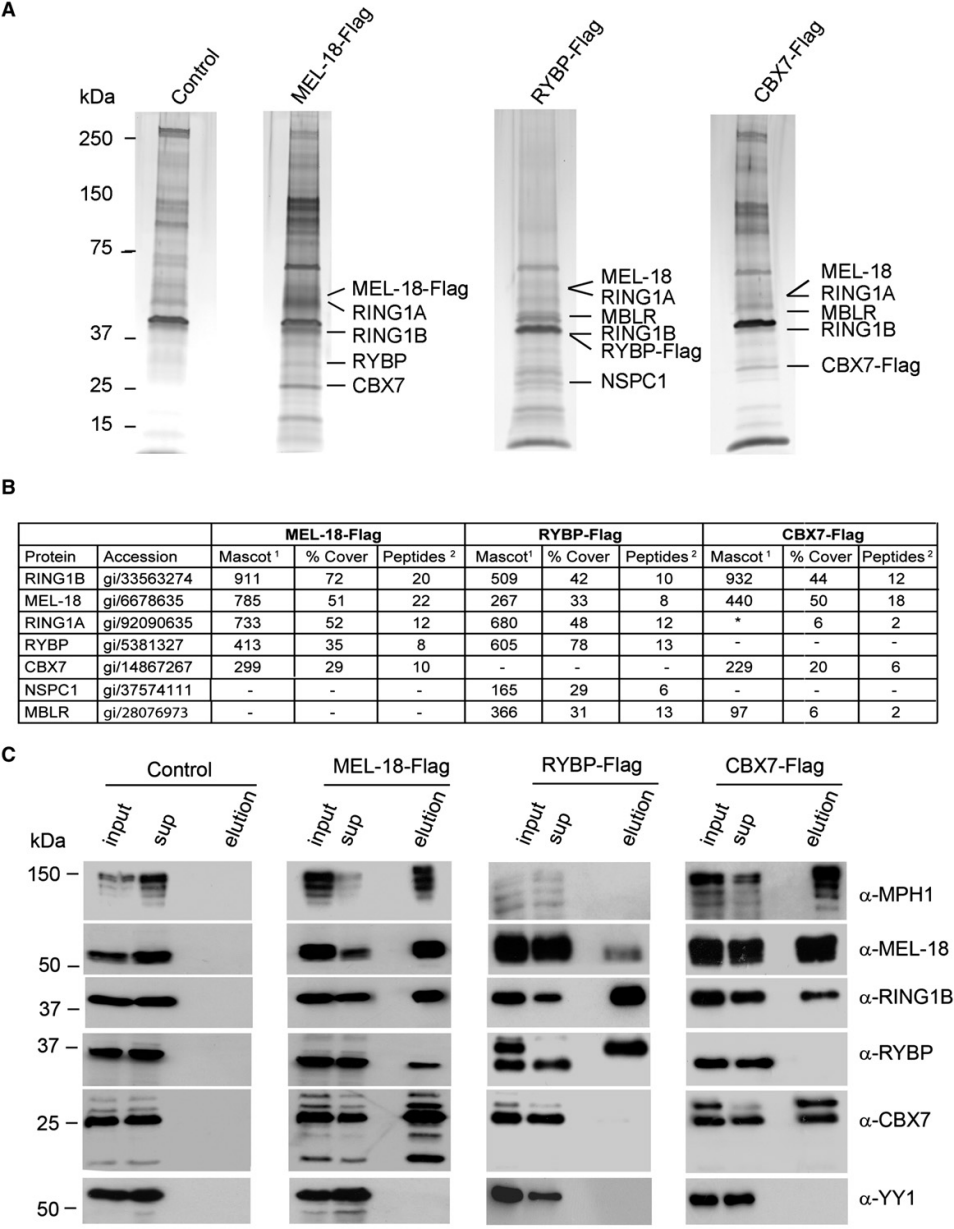
Proteomic Analysis of MEL-18, RYBP, and CBX7 Complexes in mESCs (A) Silver-stained SDS polyacrylamide gel of control, MEL-18-Flag, RYBP-Flag, and CBX7-Flag purifications. PRC1 subunits identified by mass spectrometry of excised bands are indicated. (B) Table showing the PRC1 core subunits copurifying with MEL-18-Flag, RYBP-Flag, and CBX7-Flag, as identified by mass spectrometry analysis. ^1^Mascot score for specified proteins, ^2^number of unique peptides identified. ^∗^The two peptides matched to RING1A are also present in RING1B. (C) MEL-18-Flag, RYBP-Flag, and CBX7-Flag purifications analyzed by western blot with the indicated antibodies. See also Figure S4.

**Figure S4:**
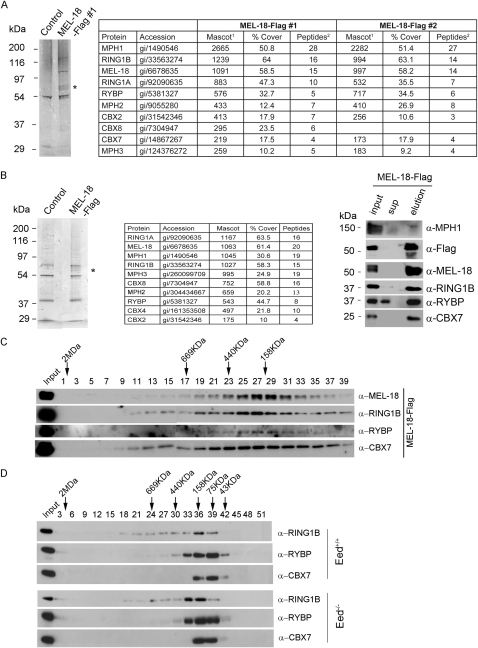
Related to Figure 4 (A) Purification of MEL-18 complexes from ESCs. Left: Colloidial Coomassie-stained SDS-polyacrylamide gel of a MEL-18-Flag purification and control purification from PGK12.1 ESCs. Asterisk indicates bands that represent the different phosphorylated forms of MEL-18 (Elderkin et al., 2007). Right: Table showing core PRC1 subunits copurifying with MEL-18-Flag, as identified by LC-MS/MS analysis in two entirely independent experiments. ^1^Mascot score for specified proteins, ^2^number of unique peptides identified. (B) Purification of MEL-18 complexes from NSCs. Left: Colloidial Coomassie-stained SDS-polyacrylamide gel of a MEL-18-Flag purification and control purification from NSCs. Asterisk indicates bands that represent MEL-18. Middle: Table showing core PRC1 subunits copurifying with MEL-18-Flag, as identified by LC-MS/MS analysis. Right: MEL-18-Flag purification analyzed by western blot with the indicated antibodies. (C) Size-exclusion analysis of mESC MEL-18 complex (25% eluted fraction) after Flag affinity purification (25% input) probed with indicated antibodies. (D) Size-exclusion analysis of nuclear extract from *Eed^+/+^* and *Eed^−/−^* ESCs probed with the indicated antibodies.

To explore the mechanism of H3K27me3-independent targeting of H2AK119u1, we carried out a proteomic screen for PRC1-associated proteins in mESCs. Because RING1B associates with non-PRC1 complexes (Gearhart et al., 2006; Ogawa et al., 2002; Sánchez et al., 2007; Trimarchi et al., 2001), we analyzed the core PRC1 protein MEL-18, a close homolog of the *Drosophila* PRC1 protein PSC that is highly expressed in mESCs (Elderkin et al., 2007) and moreover localizes to PcG target loci, albeit at low levels, in H3K27me3-deficient mESCs (Figure 1A). We established ESC lines expressing epitope-tagged MEL-18 and purified associated proteins. In liquid chromatography-tandem mass spectrometry (LC-MS/MS) experiments, we identified core PRC1 proteins, specifically RING1A/RING1B, MPH1/2/3, CBX2/7/8, and in addition, RYBP, a factor previously shown to interact with RING1A/B (Czypionka et al., 2007; Endoh et al., 2008; García et al., 1999; Wang et al., 2010) and also with the transcription factor YY1 (García et al., 1999) (Figure S4A). Identification of specific bands on silver-stained gels indicated stoichiometric amounts of PRC1 proteins and RYBP (Figures 4A, panels 1 and 2 and 4B). These findings were further substantiated by western analysis (Figure 4C, panels 1 and 2). Notably, LC-MS/MS did not detect YY1 (Figure 4B). This was confirmed by western analysis (Figure 4C and see below).

To determine whether association of MEL-18 with RYBP occurs in cells other than mESCs, we expressed MEL-18-FLAG in neural stem cells (NSCs) and again purified associated proteins (Figure S4B). LC-MS/MS/western analysis identified the major PRC1 proteins RING1A/B, CBX2/4/7/8, and MPH1 and, additionally, high levels of RYBP. Thus, association of RYBP with MEL-18 complexes is not cell type specific.

We used size-exclusion chromatography to further analyze mESC MEL-18-associated complexes (Figure S4C). Peaks for MEL-18, RING1B, RYBP, and CBX7 were centered over fractions corresponding to 150–200 kDa. A similar elution profile was observed for RING1B, RYBP, and CBX7 following size-exclusion chromatography of nuclear extracts, both from *Eed^+/+^* and *Eed* ^−/−^ mESCs (Figure S4D). These observations point to involvement of RYBP in a multiprotein complex(es) with core PRC1 proteins.

### Distinct PRC1 Complexes Defined by Mutually Exclusive Binding of RYBP or CBX7 to RING1B

**Figure 5:**
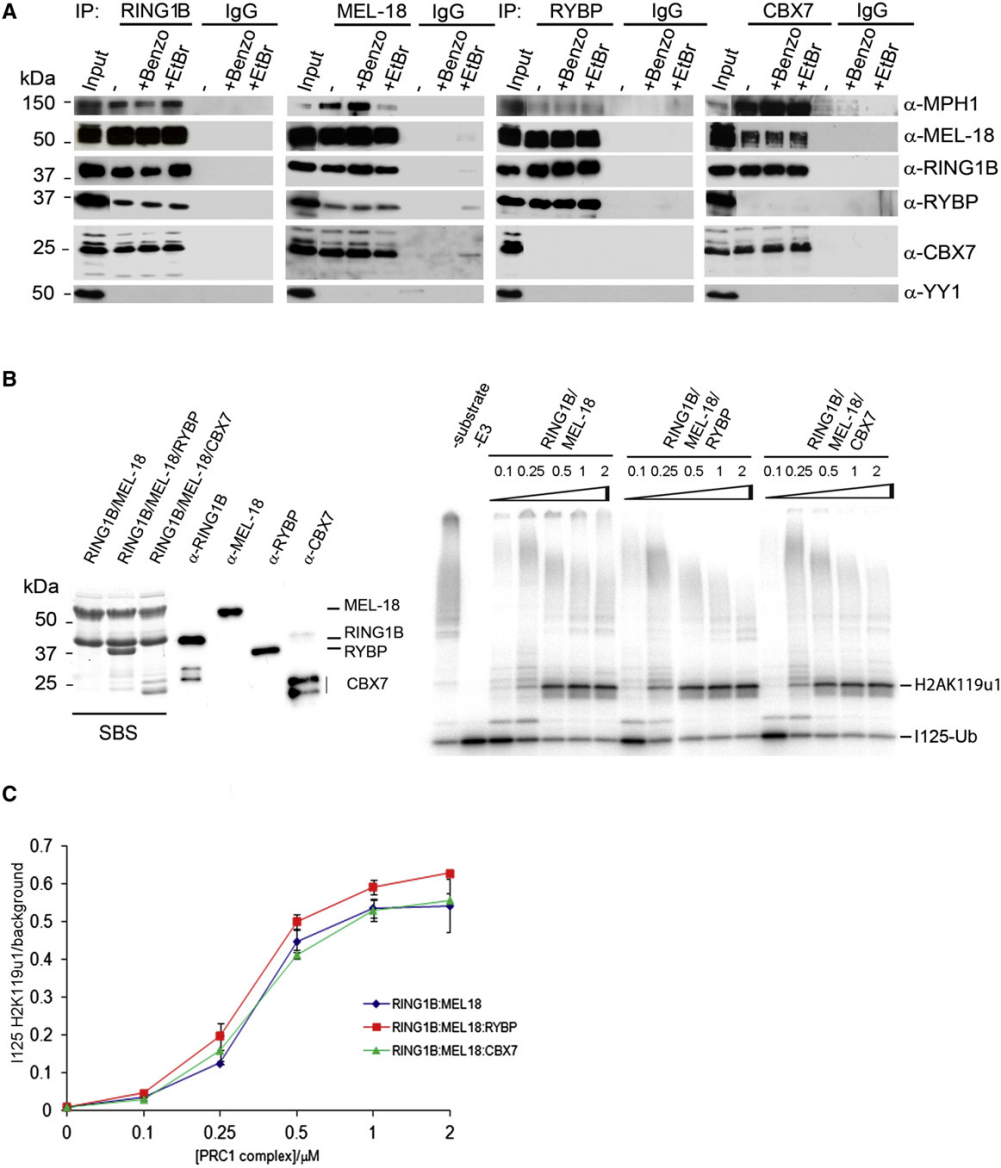
MEL-18 Interacts with RYBP and CBX7 in Mutually Exclusive Catalytically Active Complexes (A) CoIP of endogenous RING1B, MEL-18, RYBP, and CBX7 from Eed4 WT mESC nuclear extracts, analyzed by western blot with the indicated antibodies and the appropriate IgG control. Benzonase (Benzo) and ethidium bromide (EtBr) were added where indicated. 10% input and 15% of RING1B, MEL-18, RYBP, CBX7, and the appropriate control CoIP are shown. (B) Left panel: RING1B/MEL-18, RING1B/MEL-18/RYBP, and RING1B/MEL-18/CBX7 protein complexes analyzed by western blot using antibodies as indicated, or by Simply Blue Safe staining (SBS). Right panel: Ubiquitylation assays performed using indicated concentrations of RING1B/MEL-18 (lanes 3–7), RING1B/MEL-18/RYBP (lanes 8–12), and RING1B/MEL-18/CBX7 (lanes 13–17) complexes. Control assays are with substrate omitted or E3 ligase omitted. I^125^-ubiquitin-labeled products are shown. (C) Quantitation of H2AK119u1 from three independent assays as shown in (B). See also Figure S5.

**Figure S5:**
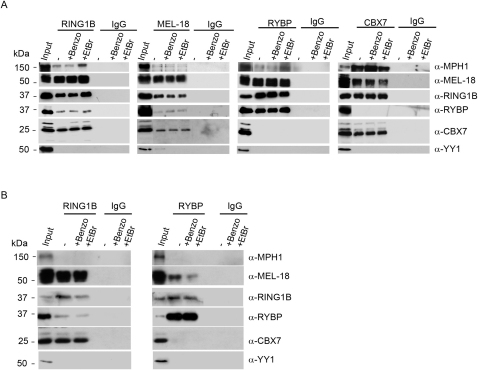
Related to Figure 5 (A) CoIP of endogenous RING1B, MEL-18, RYBP, and CBX7 and the appropriate IgG control from Eed4 cKO ESC nuclear extracts, analyzed by western blot with the indicated antibodies. Benzonase (Benzo) and ethidium bromide (EtBr) were added where indicated. 10% input and 15% of RING1B, MEL-18, RYBP, CBX7, and the appropriate control coimmunoprecipitations are shown. (B) CoIP of endogenous RING1B, RYBP and the appropriate IgG control from FSPE cell nuclear extracts, analyzed by western blot with the indicated antibodies. Benzonase (Benzo) and ethidium bromide (EtBr) were added where indicated. 10% input and 15% of RING1B, RYBP, and the appropriate control CoIP are shown.

Although RYBP has been identified as a component of E2F6 (Ogawa et al., 2002; Sánchez et al., 2007; Trimarchi et al., 2001) and BCOR (Gearhart et al., 2006; Sánchez et al., 2007), complexes that both also include RING1A/B proteins, it was not previously recognized as a component of conventional PRC1 complexes. Indeed this result is unexpected in light of structural studies that demonstrate that both RYBP and CBX proteins interact with the same surface on the RING1B protein, and that their binding is therefore mutually exclusive (García et al., 1999; Wang et al., 2010). To examine this further, and to confirm these interactions in native complexes from WT and *Eed*-deficient (Eed4 cKO) mESCs, we carried out coimmunoprecipitation (coIP) experiments with antisera to RING1B, MEL-18, RYBP, and CBX7 (Figures 5A and S5A). Immunoprecipitates were treated with either benzonase or ethidium bromide (EtBr) to confirm that interactions are not mediated by nucleic acid binding. Both RING1B and MEL-18 coIP core PRC1 proteins, i.e., RING1B, MEL-18, MPH1, CBX7, and additionally RYBP, consistent with analysis of MEL-18-Flag affinity purifications (Figure 5A, panels 1 and 2). RING1B, MEL-18, low levels of MPH1, but not CBX7 coIP with RYBP (Figure 5A, panel 3). Conversely, RING1B, MEL-18, and MPH1 but not RYBP coIP with CBX7 (Figure 5A, panel 4). No differences were observed between Eed4 WT (Figure 5A), and Eed4 cKO mESCs (Figure S5A). These results demonstrate mutually exclusive binding of CBX7 and RYBP subunits and define the existence of two distinct PRC1-like complexes comprising, on the one hand, RING1B, MEL-18, CBX7, and MPH1 and, on the other, RING1B, MEL-18, and RYBP. We refer to these complexes henceforth as CBX-PRC1 and RYBP-PRC1. CoIP experiments in a mouse fibroblast cell line also revealed mutually exclusive interaction of CBX7 and RYBP with RING1B/MEL-18 (Figure S5B), indicating that RYBP-PRC1 and CBX-PRC1 coexist in different cell types.

To further investigate the composition of CBX-PRC1 and RYBP-PRC1, we established mESC lines expressing epitope-tagged RYBP or CBX7 and then purified the associated proteins. For RYBP (Figures 4A, panel 3 and 4B), we copurified RING1A/B and MEL-18. CBX proteins, including CBX7, were not detected at all. We did, however, copurify NSPC1 and MBLR, homologs of MEL-18 that are components of the BCOR and E2F6 complexes, respectively. This finding is consistent with the previously reported association of RYBP with these complexes (Gearhart et al., 2006; Ogawa et al., 2002; Sánchez et al., 2007; Trimarchi et al., 2001). Western analysis confirmed the presence of the major components RING1A/B and MEL-18 (Figure 4C, panel 3). YY1 was not identified in RYBP-Flag immunoprecipitates, either by proteomic or by western blot analysis. Purification of epitope-tagged CBX7 identified RING1A/B and MEL-18 as major components (Figures 4A, panel 4 and 4B). Western blot analysis confirmed these associations and demonstrated presence of MPH1 (Figure 4C, panel 4). RYBP was not detected. Taken together these results substantiate that RYBP-PRC1 and CBX-PRC1 are distinct multiprotein complexes.

The activity of PRC1 complexes in H2AK119 ubiquitylation requires a minimal catalytic core comprising RING1A/B together with MEL-18/BMI-1 (Cao et al., 2005; Elderkin et al., 2007). To determine whether RYBP-PRC1, which includes both RING1B and MEL-18 subunits, functions as an E3 ligase for H2AK119u1, we reconstituted RYBP-PRC1 (RING1B, MEL-18, and RYBP) and CBX-PRC1 (RING1B, MEL-18, and CBX7) using recombinant subunits (Figure 5B, left panel) and then carried out H2A ubiquitylation assays on oligonucleosome substrate (Figure 5B, right panel). As a control, we assayed the two-component complex comprising RING1B and MEL18 that in previous studies was shown to specifically monoubiquitylate H2AK119 (Elderkin et al., 2007). All three complexes efficiently monoubiquitylated H2A (Figure 5B, right panel), and quantitative analysis demonstrated equivalent activity in all cases (Figure 5C). Thus, RYBP and CBX proteins neither stimulate nor block H2A ubiquitylation activity of PRC1 complexes in vitro.

### RYBP-PRC1 Mediates H2AK119u1 Independently of H3K27me3

**Figure 6:**
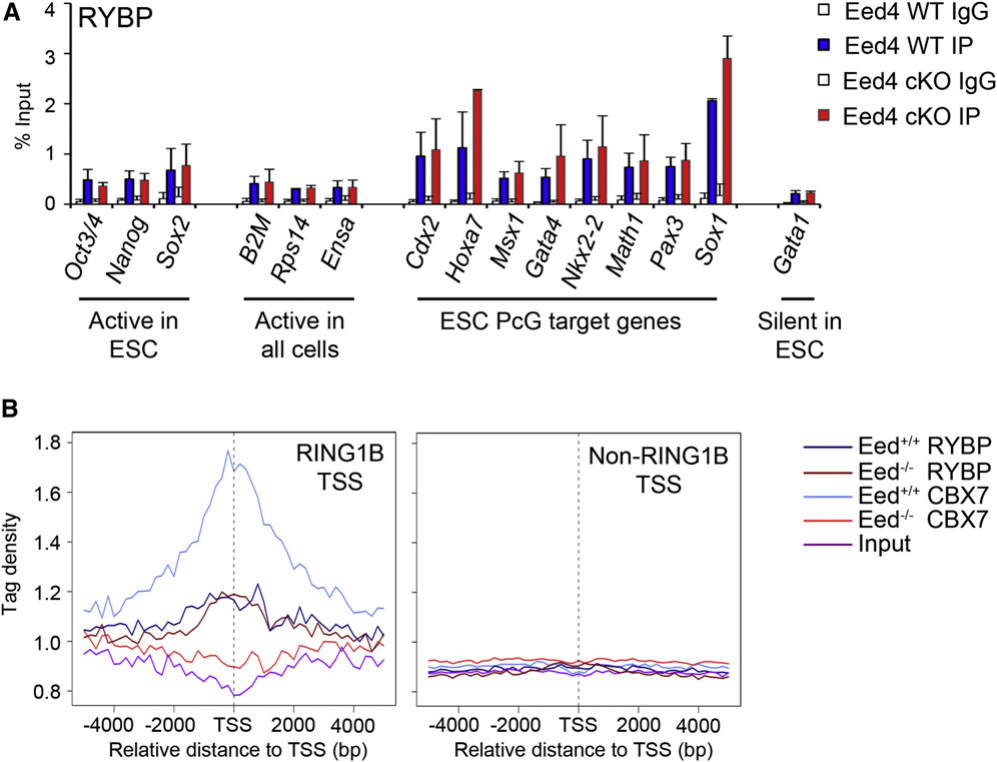
RYBP-PRC1 Is Recruited to PcG Target Genes Independently of PRC2 (A) ChIP analysis of RYBP in Eed4 WT and Eed4 cKO mESCs, showing average values + SD (n = 3). (B) Tag density across the TSS of 20 million reads randomly subsampled. RYBP, CBX7, and input tags were clustered in two different subgroups, RING1B TSS and non-RING1B TSS (see Figure 2B). Data are shown for both *Eed^+/+^* and *Eed^−/−^* mESCs. See also Figure S6.

**Figure S6:**
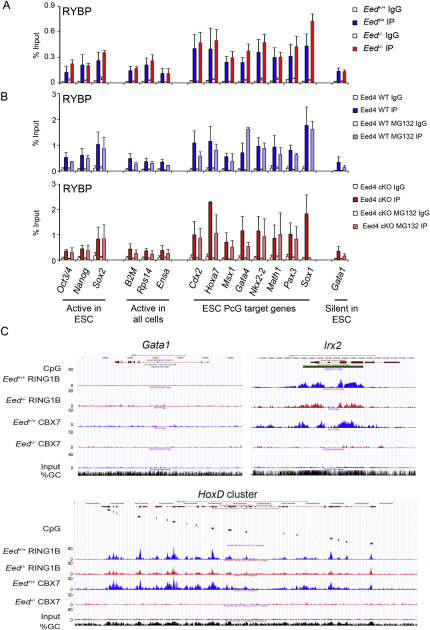
PRC1-RYBP Recruitment to PcG Target Genes in *Eed^+/+^* and *Eed^−/−^* mESCs, Related to Figure 6 (A and B) ChIP analysis of RYBP in *Eed^+/+^* compared to *Eed^−/−^* mESCs (A) and in Eed4 WT and Eed4 cKO mESCs (B) that were treated with 10 μM MG132 for 6 hr. Bars show average + SD, n = 3. (C) Example screen shots illustrate gene structure, CpG islands, and % GC tracks in addition to distribution of RING1B and CBX7 ChIP-seq reads in *Eed^+/+^* IP (blue) and *Eed^−/−^* IP (red) and input (lilac). Control gene is the non-PcG target *Gata1* (repressed). PcG targets illustrated are *Irx2* and the *HoxD* cluster.

To determine whether RYBP-PRC1 could account for H3K27me3-independent H2AK119u1, we carried out ChIP analysis for RYBP in Eed4 WT and Eed4 cKO mESCs. As shown in Figure 6A, RYBP enrichment relative to control loci was observed at several PcG target loci and, importantly, was unaffected following conditional depletion of H3K27me3 (Figure 6A). This mirrors results obtained for H2AK119u1 and contrasts with the effect on occupancy of RING1B and MEL-18 (Figure 1A). Similar results were obtained comparing constitutive *Eed*^−/−^ mESCs relative to *Eed^+/+^* controls (Figure S6A) and in MG132-treated cells (Figure S6B), the latter demonstrating that H2AK119u1 is not required for RYBP occupancy.

To confirm these findings, we carried out ChIP-seq analysis of RYBP and CBX7 occupancy in *Eed^+/+^* and *Eed*^−/−^ mESCs. Consistent with conventional ChIP analysis, we observed enrichment of RYBP over TSS of RING1B target loci, in both *Eed^+/+^* and *Eed*^−/−^ mESCs (Figure 6B, panel 1). CBX7 occupancy on the other hand was only observed in *Eed^+/+^* cells (Figures 6B, panel 1 and S6C). There was no enrichment for RYBP or CBX7 at TSS associated with non-RING1B target loci (Figure 6B, panel 2). We conclude that RYBP-PRC1 occupancy at PcG target loci is independent of H3K27me3, providing an explanation for the maintenance of H2A119u1 levels and associated target gene silencing in PRC2-deficient mESCs.

**Figure 7:**
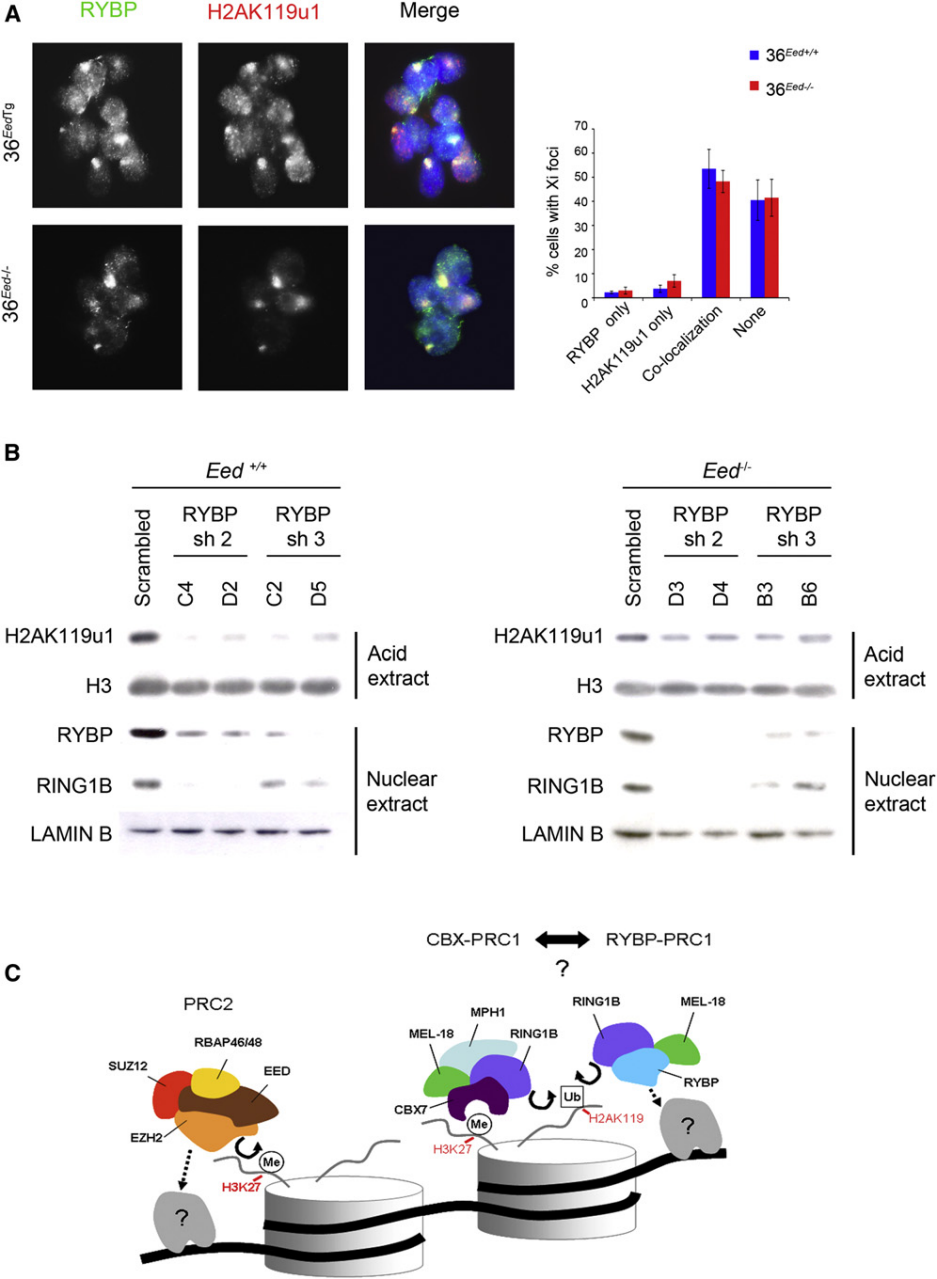
RYBP-PRC1 Is Recruited to Xist RNA Territories Independently of H3K27me3 and Is Required for H2AK119 Ubiquitylation in *Eed^+/+^* and *Eed^−/−^* mESCs (A) Immunofluorescence analysis of RYBP (green) and H2AK119u1 (red) in 36^EedTg^ and 36^Eed*−/−*^ mESCs induced to express transgenic Xist RNA. DNA was counterstained with DAPI (blue). Graphs illustrate the proportion of cells in which H2AK119u1 foci and RYBP foci colocalize, based on scoring 100 cells on each of three separate slides. (B) Stable cell lines were established following transduction of *Eed^+/+^* and *Eed^−/−^* mESCs with scrambled or either of two independent RYBP shRNAs (sh2 and sh3). Acid extracted histones (H2AK119u1 and H3) or nuclear extracts (RYBP, RING1B, and LAMIN B), were prepared and analyzed by western blot. (C) Model as discussed in text. Key: DNA (black line); nucleosomes with single N terminus of H3 and C terminus of H2A (cylinders); H3K27 trimethylation (Me); H2AK119u1 (Ub); recruitment factors (gray shape with ?). See also Figure S7.

PRC1-mediated H2AK119u1 is a marker of the inactive X chromosome (Xi) (de Napoles et al., 2004) and occurs in both the presence and the absence of H3K27me3 (Schoeftner et al., 2006). Moreover, a previous study observed RYBP localization to Xi in XX trophoblast stem cells (Arrigoni et al., 2006). To investigate whether RYBP-PRC1 could account for H3K27me3-independent H2AK119u1 on Xi, we performed RYBP immunofluorescence (IF) in mESCs that carry an autosomally located inducible *Xist* transgene on an *Eed*^−/−^ background (36^Eed−/−^) or rescued with an *Eed* transgene (36^EedTg^) (Schoeftner et al., 2006). Xist RNA territories were counterstained with antisera specific for H2AK119u1. RYBP enrichment over the Xist RNA territory was observed in both rescued and *Eed*^−/−^ mESCs (Figure 7A, left), and scoring data indicate that the levels are equivalent (Figure 7A, right). These observations suggest that RYBP-PRC1 is recruited in response to Xist RNA expression and that this accounts for H3K27me3-independent H2AK119u1 on Xi.

**Figure S7:**
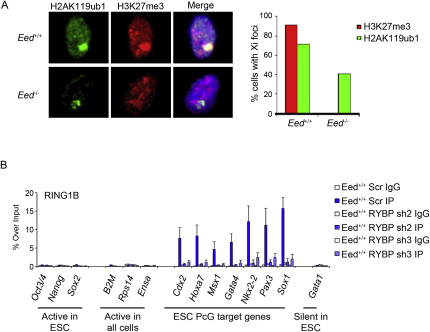
RYBP Is Required for RING1B Recruitment to PcG Target Genes, Related to Figure 7 (A) H2AK119u1 on the inactive X chromosome is retained but at diminished levels in *Eed^−/−^* embryos. IF analysis was carried out on cells from trypsin dissociated embryos recovered at E7.5. Examples of IF illustrate that H2AK119u1 Xi foci are retained in homozygous mutant XX embryos, albeit less strongly. Scoring data show that H2AK119u1 Xi foci are present in 41% of *Eed^−/−^* XX embryos (n = 243 cells from 4 embryos) compared to 72% in *Eed^+/+^* XX embryos (n = 611 cells from 6 embryos). H3K27me3 Xi foci were observed only in WT embryos. Data for heterozygous embryos are not shown but closely mirror *Eed^+/+^* embryos. (B) ChIP analysis of RING1B in wild-type (*Eed^+/+^*) mESCs transduced with scrambled (Scr) or either of two independent RYBP shRNAs (sh2 and sh3). Bars show average + SD, n = 3.

To directly test the role of RYBP in maintaining levels of H2AK119u1, we analyzed *Eed^+/+^* and *Eed*^−/−^ mESCs in which we expressed one of two different shRNA hairpins to deplete RYBP levels (Figure 7B). In *Eed^+/+^* mESCs, RYBP knockdown led to substantially reduced levels of H2AK119u1 (Figure 7B, left). A smaller effect was observed with *Eed*^−/−^ mESCs (Figure 7B, right), although it was difficult to derive stable undifferentiated mESCs in this case, indicating selection for mESCs in which RYBP depletion is ineffective. Interestingly, we observed a marked reduction in the levels of RING1B in RYBP knockdown cells (Figure 7B), suggesting that RYBP is important for the stabilization of RING1B. A similar effect is seen in cells depleted for PRC2 core components (Pasini et al., 2007; Silva et al., 2003). In line with reduced global levels of RING1B, we observed reduced occupancy of RING1B at PcG target loci (Figure S7B). In sum, these data demonstrate a central role for RYBP in mediating H2AK119u1.

## Discussion

### RYBP-PRC1 Polycomb-Repressive Complex

RYBP was first identified in a yeast two-hybrid screen for RING1A and was further shown to interact with the transcription factor YY1 (García et al., 1999). The latter finding is consistent with a previous study that identified YAF2, a close homolog of RYBP, as a YY1 interactor (Kalenik et al., 1997). Subsequent studies identified RYBP as having a role in apoptosis (Zheng et al., 2001). At present it is not clear whether these different ascribed functions and associations can be reconciled or whether RYBP is in fact multifunctional and participates in distinct pathways, interacting with different factors. Genetic studies in mouse demonstrate that RYBP is essential for early embryogenesis (Pirity et al., 2005), and close homologs are found in many species. Interestingly, RYBP mutations in *Drosophila* do not give a Polycomb phenotype (Bejarano et al., 2005). The presence in vertebrates of YAF2 adds further complexity to considerations of function.

Given the known interaction of RYBP with RING1A/B, copurification with PRC1 proteins was on one level unsurprising. Indeed RYBP/YAF2 has been identified as a component of BCOR (Gearhart et al., 2006; Sánchez et al., 2007) and E2F6 complexes (Sánchez et al., 2007; Trimarchi et al., 2001), together with RING1B and the mammalian PSC homologs NSPC1 and MBLR, respectively (Ogawa et al., 2002; Sánchez et al., 2007). However, homologs of other *Drosophila* PRC1 core subunits, PH and PC, were absent in these purifications, and moreover a number of independent analyses of PRC1 have not identified RYBP (Cao et al., 2005; Eskeland et al., 2010; Maertens et al., 2009; Saurin et al., 2001; Shao et al., 1999; Wang et al., 2004). In some cases this can be attributed retrospectively to the use of strategies based on epitope tagging of CBX proteins (Maertens et al., 2009; Ren and Kerppola, 2011; Saurin et al., 2001). In other instances, however, purifications were based on an H2A ubiquitylation activity assay (Wang et al., 2004) or epitope tagging of other PRC1 components (Cao et al., 2005; Eskeland et al., 2010; Maertens et al., 2009; Saurin et al., 2001; Shao et al., 1999; Wang et al., 2004). Notably, RYBP was not identified in previous purifications of PRC1 via epitope-tagged MEL-18 (Elderkin et al., 2007; Maertens et al., 2009). We assume this is attributable to technical factors as our analysis demonstrates that RYBP-PRC1 is present in mESCs and in different cell types. BMI-1, a second PSC homolog present in conventional PRC1 complexes, also copurifies stoichiometric levels of RYBP in mESCs and mouse NSCs (mNSCs) (data not shown), supporting a wider significance of RYBP-PRC1 complexes.

The fact that we see reciprocal immunoprecipitation of RYBP and MEL-18 in native extracts confirms that this interaction is physiologically relevant. Importantly, we show that the RYBP-PRC1 complex comprising RING1B, MEL-18, and RYBP can monoubiquitylate H2A on nucleosome substrates in vitro and that the activity of the complex is equivalent to that of CBX-PRC1.

In line with prior evidence (García et al., 1999; Wang et al., 2010), we find that interaction of RYBP and CBX7 with RING1B is mutually exclusive. We assume that the same is true for other mammalian homologs of *Drosophila* PC, specifically CBX2, 4, 6, and 8, but this has not been tested. We also observed that RYBP-PRC1 has a reduced association with MPH1. Consistent with this, RING1B but not MPH1/2 localizes to Xist RNA territories in *Eed*-deficient mESCs (Schoeftner et al., 2006). We assume that RYBP-PRC1 excludes other mammalian homologs of *Drosophila* PH, MPH2 and MPH3, but this also is untested. PH has been reported to interact with PSC (Kyba and Brock, 1998), and therefore to explain near exclusion of MPH1 from RYBP-PRC1, we speculate that RYBP occludes the required interaction surface on MEL-18.

Collectively our observations indicate that RYBP-PRC1 is comprised of three core components, RYBP, RING1B, and MEL-18/BMI-1. Our proteomic studies using epitope-tagged MEL-18 (this study) and BMI-1 (not shown) did not reveal other stoichiometric components, although we cannot rule out the presence of other key components at substoichiometric levels. Additionally, although in mESCs MEL-18 is an abundant PSC homolog, we cannot rule out a significant contribution of BMI-1 or other PSC homologs to RYBP-PRC1. Indeed, mESC RYBP complexes included the PSC homologs NSPC1 and MBLR, associated with BCOR and E2F6 complexes, respectively (Gearhart et al., 2006; Ogawa et al., 2002; Sánchez et al., 2007; Trimarchi et al., 2001). Studies in *Drosophila* have shown that the PRC1-related complex dRAF, comprised of RING1, PSC, and KDM2, plays a central role in global H2AK119u1 (Lagarou et al., 2008). This provides an interesting parallel with mESCs in which the BCOR complex includes a mammalian homolog of KDM2, KDM2B (Gearhart et al., 2006; Sánchez et al., 2007). However, there is no evidence that RYBP in *Drosophila* participates in PRC1-related complexes.

### Recruitment of RYBP-PRC1

Genome-wide analysis of RING1B binding in the absence of H3K27me3 indicates that RYBP-PRC1 and PRC2 are recruited to many of the same target genes. It is intriguing that distribution of RING1B, localizing across CpG islands, resembles that of PRC2, even in the absence of H3K27me3. Indeed a recent study has suggested that unmethylated CpG domains may be sufficient to recruit PRC2 (Mendenhall et al., 2010). Although it is possible that the same signature recruits RYBP-PRC1, our analysis demonstrates sites bound by RING1B only in the presence of H3K27me3 and other sites where H3K27me3 is less important for RING1B targeting. Collectively, these observations suggest some differences in the targeting mechanisms of PRC2 and RYBP-PRC1, or at least in the relative contribution of the two pathways at specific loci.

Given that RYBP-PRC1 and PRC2 have significantly overlapping targets, can RYBP provide clues as to how targeting is mediated? As discussed above, RYBP was previously shown to interact with the transcription factor YY1 (García et al., 1999). Interestingly YY1 is the mammalian homolog of *Drosophila* PHO, which in the context of the PHO-RC complex plays a central role in PcG targeting (Klymenko et al., 2006). However, we did not find YY1 together with PRC1 in proteomic or native immunoprecipitation analyses. Moreover mapping of YY1-binding sites in mESCs reveals no significant overlap with PRC2 binding (Squazzo et al., 2006). We therefore conclude that interaction of RYBP with YY1 is unlikely to be relevant, at least in mESCs.

RYBP has a single conserved domain, a Ranbp2 zinc finger (Ranbp2-ZnF). A subset of proteins with this domain are associated with RNA metabolism, and moreover, nuclear magnetic resonance (NMR) studies have demonstrated that the Ranbp2-ZnF in these proteins binds RNA (Nguyen et al., 2011). This is potentially interesting in light of recruitment of RYBP in response to Xist RNA expression and also a series of recent studies suggesting a wider role for noncoding RNA in PcG recruitment (reviewed in Pauli et al., 2011). Arguing against this, comparative analysis indicates that the RYBP Ranbp2-ZnF belongs to a different class and that none of the contact residues for RNA binding are conserved or similar (not shown). A further subset of Ranbp2-ZnF proteins interact with ubiquitin, also characterized at the structural level (Wang et al., 2003), and here RYBP does show greater similarity. Indeed, it has been suggested that RYBP interacts with H2AK119u1 and additionally is subject to self-monoubiquitylation as a consequence of being in complex with RING1B (Arrigoni et al., 2006). This could be argued to point to a role for RYBP-PRC1 in maintaining H2AK119u1 by interacting with pre-existing marks on neighboring nucleosomes. However, our observation that RYBP is not displaced and that H2AK119u1 can be re-established following depletion by MG132 treatment, in both the presence and the absence of H3K27me3, appears to discount this idea. In sum, involvement of RYBP provides some intriguing clues that may help to understand PcG targeting, but further studies are needed to determine which, if any, are relevant.

### Interplay of PRCs

Our data suggest that parallel pathways target H2A ubiquitylation to PcG targets in mESCs and on the inactive X chromosome. A model illustrating this is shown in Figure 7C. As discussed, the primary signal that recruits PRC2 and RYBP-PRC1 is unknown. CBX-PRC1 recruitment, on the other hand, is linked to PRC2-mediated H3K27me3. Although RYBP-PRC1 recruitment can occur in the absence of H3K27me3, we cannot rule out that CBX-PRC1 binding is at least partially dependent on RYBP-PRC1. In support of this view, we observed significant reduction of H2AK119u1 following RYBP knockdown in *Eed^+/+^* mESCs. Linked to this point, it is notable that reduced occupancy of RING1B and MEL-18 in PRC2-deficient mESCs is not mirrored by a greater reduction in global and local H2AK119u1 levels. One possible explanation is that RYBP-PRC1 has a short residence time on chromatin (relative to CBX-PRC1), sufficient for catalysis but not for efficient formaldehyde crosslinking, as has been observed for the interaction of the methylated DNA-binding protein MeCp2 with meCpG (Schmiedeberg et al., 2009).

Parallel targeting of RYBP-PRC1 and CBX-PRC1 provides an explanation for the fact that PRC2 null mESCs self-renew and retain pluripotency (Faust et al., 1998; O'Carroll et al., 2001; Pasini et al., 2004), whereas RING1A/B double-knockout cells (PRC1 null) cannot self-renew and therefore differentiate (Endoh et al., 2008). This may be a somewhat simplistic interpretation as mESCs lacking the PRC2 subunit SUZ12 (Pasini et al., 2004), and also cells in which both RING1B and EED are deleted (Leeb et al., 2010), show aberrant differentiation phenotypes. Moreover, recent studies have shown that depletion of JARID2, a PRC2-associated cofactor, blocks differentiation of mESCs (reviewed in Herz and Shilatifard, 2010), as does *Jarid2* deletion (Landeira et al., 2010).

The fact that we see involvement of RYBP-PRC1 in Xist RNA-mediated silencing suggests a wider role for this complex. In future studies it will be interesting to determine whether RYBP has a role in other instances of H3K27me3-independent recruitment of PRC1 complexes, for example on paternal chromosomes in early preimplantation mouse embryos (Puschendorf et al., 2008).

## Experimental Procedures

### Cell Culture

mESCs, FSPE fibroblasts, and NSCs were cultivated using established methods. Stable lines expressing Flag-tagged proteins were produced by lipofection followed by selection for antibiotic resistance. Full details are provided in the Extended Experimental Procedures. Pluripotency assays were performed using the alkaline phosphatase detection kit (Millipore).

### ChIP and ChIP-Seq

ChIP was performed essentially as described (Stock et al., 2007) with some modifications used for specific antibodies, as detailed in the Extended Experimental Procedures. Results were analyzed either by qPCR or ChIP-seq as indicated. ChIP-seq was either by single- or paired-end methods as detailed in the Extended Experimental Procedures. Tags were mapped using bowtie (Langmead et al., 2009) excluding nonunique mappings (-m 1). Following alignment to the mouse genome (mm9), data were visualized on UCSC (Kent et al., 2002) and GBrowse (Stein et al., 2002). Single and paired-end tags were mapped on GBrowse, and peak identification was performed with MACS (Zhang et al., 2008) with a false discovery rate (FDR) < 2% and number of tags in the peak > 100. Nearest gene and overlaps to location used Cisgenome (Ji et al., 2008) and custom scripts. Peak intersection analysis used intersectBed (Quinlan and Hall, 2010) with CpG island data downloaded from UCSC mm9 table browser. Average profile across TSS used CEAS (Shin et al., 2009) after normalizing by random subsampling. For RYBP and CBX7 datasets, random subsampling was applied to RING1B and non-RING1B peaks selected from RING1B ChIP-seq peak list in the *Eed^+/+^* sample. Tag density analysis was performed with sitepro, part of the CEAS package, (Shin et al., 2009). Full details are provided in the Extended Experimental Procedures.

### Western Blot Analysis

Levels of histone modifications and nonhistone protein were determined by western blot analysis of acid-extracted proteins or nuclear extracts, respectively, using appropriate primary and secondary antibodies as detailed in the Extended Experimental Procedures.

### Gene Expression Analysis

Expression levels of PcG target genes were determined by qRT-PCR using standard methods.

### Biochemical Analysis

Purifications from nuclear extract were carried out as described previously (van den Berg et al., 2010). Protein identification was by LC-MS/MS on an LTQ Orbitrap Velos Mass spectrometer. Data were searched against Uniprot 2011.03 using Mascot software.

Size-exclusion chromatography was carried out on a Superose 6 gel filtration column as detailed in the Extended Experimental Procedures. Recombinant complexes comprising combinations of full-length MEL-18, RING1B, RYBP, and CBX7 were purified from Sf9 cells essentially as described (Elderkin et al., 2007), using the Bac-to-Bac system (Invitrogen). Full details are provided in the Extended Experimental Procedures. Ubiquitylation assays were performed as described previously (Elderkin et al., 2007) and as detailed in the Extended Experimental Procedures. Immunoprecipitations were from nuclear extracts (Dignam et al., 1983) using appropriate primary and secondary antibodies either without treatment or in the presence of benzonase or *EtBr* as described previously (van den Berg et al., 2010).

### Immunofluorescence

Immunofluorescence (IF) was performed as described (de Napoles et al., 2004; Fang et al., 2004) with antibody dilutions, as detailed in the Extended Experimental Procedures. Images were acquired on a Zeiss AX10 microscope equipped with AxioCam MRm CCD camera using AxioVision software.

Extended Experimental Procedures **Embryos and Cell Culture**

Embryonic day (E) 7.5 XX embryos for immunofluorescence analysis were obtained from timed matings and processed as described previously (de Napoles et al., 2004; Silva et al., 2003). Eed4 cKO (Ura et al., 2008), ES-ERT2 *Ring1A*^−/−^ (Endoh et al., 2008) and PGK12.1 (Mak et al., 2002) ESCs were cultured as described previously (Nesterova et al., 2008). *Eed^+/+^*, *Eed*^−/−^, 36^EedTg^, and 36^Eed−/−^ (Schoeftner et al., 2006) mESCs were cultured in KO DMEM supplemented with 10% FCS, 5% KO serum replacement (Invitrogen), Leukemia-Inhibitor factor, 50 μg/ml penicillin/streptomycin, 2 mM L-glutamine, 1x nonessential amino acids, and 50 μM 2-mercaptoethanol. Eed4 cKO were grown in 0.1% gelatin-coated dishes. For *Eed^+/+^*, *Eed*^−/−^, and ES-ERT2 *Ring1A*^−/−^ cells, dishes were coated with 0.1% gelatine and mitomycin-C inactivated primary embryonic fibroblasts (PEFs). Eed4 cKO were treated for 15 days with 1 μg/ml of doxycycline. Conditional deletion of *Ring1B* from ES-ERT2 *Ring1A*^−/−^ cells was carried out as previously described (Stock et al., 2007). MG132 treatment of Eed4 and Eed4 cKO was for 6 hr with 10 μM MG132. *Eed^+/+^* and *Eed*^−/−^ ESCs were trypsinized and pre-plated for 30 min in order to remove contaminating feeder cells prior to harvesting. 36^EedTg^ and 36^Eed−/−^ were treated for 48 hr with 1 μg/ml of doxycycline. mNSCs were generated and cultured as described previously (Conti et al., 2005) in N2B27 medium (Stem Cell Sciences) supplemented with 10 ng/ml human fibroblast growth factor (FGF) and 10 ng/ml murine epidermal growth factor (EGF) (Preprotech). The FSPE mouse fibroblast cell line (Mermoud et al., 1999) was cultured in EC10 medium (DMEM suplemented with 10% FCS 50 μg/ml penicillin/streptomycin, 2 mM L-glutamine, 1x nonessential amino acids, and 50 μM 2-mercaptoethanol).

**Generation of Stable MEL-18-Flag, RYBP-Flag, and CBX7-Flag Cell Lines**

MEL-18 full-length cDNA was amplified from PGK12.1 mESC mRNA using melF (GGATCCACCATGCATCGGAC) and melR (GCTAGCAGGGGGGCAAGGAGCGCCATTAACA) primers and cloned into pCBA-2xFlag (van den Berg et al., 2008). RYBP full-length cDNA was amplified from the mouse RYBP cDNA image clone ID 8861345 (Source Bioscience) using rybpF (GGATCCATGACCATGGGCGACAAGAAGA) and rybpR (GCTAGCGAAAGATTCATCATTCACTGCT) and cloned into pCBA-2xFlag. MEL-18 and RYBP full-length cDNA fused to a C-terminal 2xFlag was amplified form pCBA-2xFlag vector using universal forward (CTCGAGGCGAGAGAGGATCCACCATG) and reverse (GCGGCCGCCTTTCACTTATCGTCGTCATCCTT) primers and cloned into pCAG vector (Elderkin et al., 2007). CBX7 full-length cDNA was amplified from PGK12.1 mESC mRNA using cbxF (GGATCCACCATGGAGCTGTCAGCCATAGGCGAGCAGG) and cbxR (GGTACCCAGCTTCTCGTTGCGGTCTCGGAA) primers and cloned into pCBA-2xFlag (van den Berg et al., 2008). MEL-18 and CBX7 pCBA and RYBP pCAG vectors were transfected into PGK12.1 ESCs with lipofectamine 2000 (Invitrogen) according to manufacturer's instructions. Flag-tagged MEL-18 and CBX7 stably expressing cells were selected by growth in G418 (400 μg/ml GIBCO). Flag-tagged RYBP stably expressing cells were selected by growth in puromycin (1.7 μg/ml). Stable NSC lines expressing Flag-tagged MEL-18 were generated using an Amaxa Nucleofector and Nucleofector Kit V (Lonza) followed by selection with puromycin (500 ng/ml).

**Chromatin Immunoprecipitation**

ChIP experiments were performed as described (Stock et al., 2007) with some modifications. Two hundred micrograms of chromatin was used for each IP and the antibodies used were H2AK119u1 (05-678, Millipore) 50 μl, RING1B (Atsuta et al., 2001) 15 μl, H3 (ab1791, Abcam) 3 μl, MEL-18 (sc-10744, Santa Cruz) 30 μl, H3K27me3 (CS-069-100, Diagenode) 5 μl, IgG antibody (M7023, Sigma) 2 μl, was used as a control except for H2AK119u1 where a no antibody sample was used. ChIP DNA was dissolved in 150 μl 1x TE and 5 μl was used per real time PCR reaction. RT-PCR was carried out using SYBR (Bio-Rad), following manufacturer's instructions. Enrichment was normalized to H3 or input DNA as stated. Primer sequences are given below.

ChIP for RING1B, EZH2, RYBP, CBX7, H3, and H3K27me3 were carried out using a different protocol. Cells (1–2 x 10^8^ cells) were fixed in PBS with 2 μM EGS (21565, Thermofisher) for 1 hr at room temperature (RT). After washing, cells were fixed again in ChIP fix buffer (1% formaldehyde, 5 μM EGTA, 10 μM EDTA, 1 mM NaCl, and 0.5 mM HEPES in PBS) for 10 min at RT. Fixation was stopped by glycine to a final concentration of 125 mM. Cell extracts were lysed (1% SDS, 10 mM EDTA pH 8.0, 50 mM Tris-HCl, pH 8.1) with complete protease inhibitors (Roche), and sonication was performed using a BioRupter sonicator (Diagenode) to obtain an average DNA fragment size of 300 bp. Chromatin was diluted with 1% Triton X-100, 2 mM EDTA, pH 8.0, 150 mM NaCl, 20 mM Tris-HCl, pH 8.1 containing complete protease inhibitors. Protein G Sepharose and rProtein A Sepharose (17-0618-01 and 17-1279-01, respectively, GE Healthcare) were blocked for 1 hr at 4°C with 1 mg/ml BSA and 1 mg/ml yeast tRNA (R8759-500UN, Sigma). One hundred and forty micrograms of chromatin was used for each IP and the antibodies were RING1B (Atsuta et al., 2001) 15 μl, EZH2 (pAb-039-050, Diagenode), 5 μl, H3 (ab1791, Abcam) 3 μl, RYBP (AB3637, Millipore) 5 μl, CBX7 (ab21873, Abcam), 5 μl and IgG (M7023, Sigma) 2 μl. Chromatin was precleared with blocked beads for 1 hr at 4°C and incubated with antibodies overnight at 4°C with rotation. Protein-antibody complexes were pulled down by adding beads to the solution for 2 hr. Complexes were washed 4 times with 1% SDS, 1% Triton X-100, 2 mM EDTA pH 8.0, 150 mM NaCl and 200 mM Tris-HCl, pH 8.0, followed by 1 wash in 0.1% SDS, 1% Triton X-100, 500 mM NaCl and 20 mM Tris-HCl pH 8.1. The last wash was in TE. Samples were treated with RNase A and Proteinase K and reverse crosslinked overnight. DNA was then purified using a PureLink PCR micro column (Invitrogen). DNA was used for ChIP-seq or diluted in H_2_O to a final volume of 150 μl and 5 μl was used per real-time PCR reaction. RT-PCR was carried out using SYBR (Bio-Rad), following manufacturer's instructions. Enrichment was normalized to input DNA. Primer sequences are given below.

**ChIP-Seq**

For single-end ChIP-seq, DNA was end-repaired, A-tailed, and adaptor-ligated using a ChIP-seq DNA Sample prep Kit (Illumina). A QIAGEN PCR purification step was performed following each enzymatic reaction. The adaptor-ligated material was then PCR amplified, using 18 cycles of PCR before size selection of 200–500 bp fragments on an agarose gel. The library was then extracted using a Qiaquick gel extraction kit and checked for concentration and integrity. DNA was sequenced using an Illumina Genome Analyzer II. To give more depth to the analysis, three lanes of single-end ChIP-seq were used for the *Eed*^−/−^ sample and input and one lane for *Eed^+/+^* ESCs.

Paired-end ChIP-seq libraries (CBX7 and RYBP) were constructed using the NEBNext DNA Sample Prep Master Mix Set 1 Kit (NEB) with the following modifications: end Repair was performed with 1 μl of enzyme mix and adapters were ligated using 4 μl of ligase. Multiplexing Sample Preparation Oliogonucleotide Kit was used to amplify the library with the following primers:

5′-AATGATACGGCGACCACCGAGATCTACACTCTTTCCCTACACGACGCTCTTCCGATCT-3′ and Index primer 5′-CAAGCAGAAGACGGCATACGAGAT[INDEX]CAGTGACTGGAGTTCAGACGTGTGCTCTTCCGATCT-3′). Indexes were according to the six base tags developed by Illumina.

The amplified library was purified using Ampure Xp beads and size distribution determined using a Tapestation 1DK system (Agilent). The relative concentration of the libraries was determined by real-time PCR using Agilent QPCR Library Quantification Kit. Finally, a second real-time was performed to measure the relative concentration of the pool compared to a previously sequenced ChIP library in order to determine the volume to use for sequencing on an Illumina HiSeq2000. Final datasets for single-end ChIP-seq were 16,814,546 mapped tags for the *Eed^+/+^* RING1B sample, 52,569,530 input, 70,343,706 for the *Eed*^−/−^ RING1B. Final datasets for paired-end ChIP-seq were 42,833,892 mapped tags for the *Eed^+/+^* CBX7 sample, 24,070,216 for the *Eed*^−/−^ CBX7, 28,009,124 for the input, 34,995,004 mapped tags for the *Eed^+/+^* RYBP, and 29,324,450 mapped tags for the *Eed*^−/−^ RYBP. Complete datasets for RING1B and CBX7 can be viewed at http://genome.ucsc.edu/cgi-bin/hgTracks?hgS_doOtherUser=submit&hgS_otherUserName=Staylor%40molbiol.ox.ac.uk&hgS_otherUserSessionNamemm9_ring1b. Tables with MACS peak analysis are available on request.

Peak intersection analysis used intersectBed (Quinlan and Hall, 2010) between *Eed^+/+^* and *Eed*^−/−^ RING1B peaks. Additionally we compared our RING1B peak data with previously published data for RING1B and H3K27me3 (Ku et al., 2008), comprising approximately 1,500 significantly enriched genomic sites for RING1B that in large part overlapped with H3K27me3 peaks. Of the 1,500 RING1B peaks reported 1,485 overlap with peaks identified in this study. That we see some additional peaks is a reflection of improved HT sequencing technology providing a larger numbers of reads.

**Gene Ontology Functional Analysis**

Gene ontology enrichment was carried out to compare *Eed^+/+^* with *Eed*^−/−^ peaks using DAVID gene ontology functional annotation tool (Dennis et al., 2003; Huang et al., 2009). Bonferroni correction for multiple hypotheses was used for correcting p values.

**Extracts, Western Blot Analysis, and Antibodies**

For nuclear extracts for western analysis and size-exclusion chromatography, cells were harvested, washed with PBS, resuspended in Buffer H (20 mM HEPES, pH 7.9, 1.5 mM MgCl_2_, 10 mM KCl, 1 mM DTT) and plasma membrane was then disrupted by passing cells through a 27 G needle. Nuclei were spun down (5 min x 13,000 rpm) and resuspended in Buffer D (20 mM HEPES, pH 7.9, 1.5 mM MgCl_2_, 0.2 mM EDTA, 0.42 M KCl, 20% glycerol, and 1 mM DTT). Extraction of nuclear proteins was performed at 4°C for 2 hr with vigorous shaking. The nuclear fraction was cleared by centrifuging for 30 min at 13,000 rpm.

For acid extraction of histones cells were washed in RSB buffer (10 mM HCl, pH 7.4, 10 mM NaCl, 3 mM MgCl_2_), then lysed in RSB plus 0.5% NP-40 for 10 min on ice. Histone extraction was performed by resuspending the nuclear pellet in 0.5 ml 5 mM MgCl_2_ and 0.5 ml 0.8 M HCl and incubating for 20 min on ice. Supernatant was obtained by centrifuging for 20 min at 13,000 rpm followed by precipitation of histones by cooling on ice for 30 min after addition of TCA to a final concentration of 25%. Histones were pelleted (15 min x 13,000 rpm), washed twice with acetone and resuspended in 1 x SDS loading buffer.

For western blots blocking was in either TBS, pH 8, with 5% milk, TBS, pH 8, with 0.1% Tween-20 and 5% milk or PBS with 0.1% Tween-20 and 5% milk; primary antibody incubation was in TBS, pH 8, TBS, pH 8 with 0.1% Tween-20 and 5% milk or PBS with 0.1% Tween-20 and 5% milk overnight; secondary antibody incubation was in TBS, pH 8, with 5% milk, TBS, pH 8, with 0.1% Tween-20 and 5% milk or PBS with 0.1% Tween-20 and 5% milk for 1 hr and washes were in TBS, pH 8, TBS, pH 8 with 0.1% Tween-20 or PBS with 0.1% Tween-20. Primary antibodies used were H2AK119u1 (05-678, Millipore) 1:500, H3K27me3 (CS-069-100, Diagenode) 1:1000, H3 (ab1791, Abcam) 1:10000, RING1B (Atsuta et al., 2001) 1:500, MEL-18 (ab5267, abcam) 1:500, RYBP (AB3637, Milipore) 1:500, CBX7 (07-981, Milipore) 1:500, MPH1 (Miyagishima et al., 2003) 1:10, SUZ12 (07-689, Millipore) 1:1000, EZH2 (pAb-039-050, Diagenode) 1:750, EED (Sewalt et al., 1998) 1:650, Flag (F1804, Sigma) 1:1000, CBX7 (ab21873, Abcam) 1:1000, and LAMIN B (sc-6216 and sc-6217, Santa Cruz) 1:1000. Secondary antibodies were either sheep anti-mouse IgG HPR linked (GE Healthcare) 1:2000, donkey anti-rabbit IgG HRP linked (GE Healthcare) 1:20000, or donkey anti-goat IgG HRP linked (Santa Cruz) 1:2000.

**MEL-18-Flag, RYBP-Flag, and CBX7-Flag Purification and Mass Spectrometry**

Purification of MEL-18-Flag, RYBP-Flag, and CBX7-Flag from nuclear extract was carried out as described previously (van den Berg et al., 2010). Eluted control and MEL-18-Flag purifications were TCA precipitated, proteins were separated by polacrylamide gel electrophoresis and were either stained with Colloidal Blue Staining Kit (Invitrogen) and analyzed by mass spectrometry as described previously (van den Berg et al., 2010) or stained with Silver QuestTM (Invitrogen), with unique bands in the MEL-18-Flag purification and the corresponding region in the control lane being excised and analyzed by mass spectrometry. Briefly one-half of each excised band was reduced, carbamidomethylated, and digested overnight with trypsin 16 (Promega sequencing grade, 10 ng/μl in 25 mM ammonium bicarbonate). 1/5 of each of the resulting tryptic digests was analyzed by LC-MS/MS. LC separation was achieved on a reversed-phase column (Reprosil C18AQ, 0.05 × 150 mm, 3 μm particle size), with an acetonitrile gradient (4%–40% over 30 min, containing 0.1% formic acid, at a flow rate of 120 nl/min). The column was coupled via a nanospray ion source (Proxeon) to an LTQ Orbitrap Velos mass spectrometer (Thermo) operated in data-dependent acquisition mode. The acquisition cycle consisted of a high-resolution precursor ion spectrum over the m/z range 350–1500, followed by up to 20 CID spectra (with a 60 s dynamic exclusion of former target ions). Mass spectrometric data were searched against the mammalian entries in Uniprot 2011.03 using Mascot software (Matrix Science) and the search results were processed using Scaffold software (Proteome Software Inc). Criteria for protein identification were: minimum of two peptides, each with a probability of >80% and an overall protein probability of >95%.

**Size Exclusion**

MEL-18-Flag purifications were carried out as described previously (van den Berg et al., 2010) except complexes were eluted overnight. Eluted complexes were concentrated using a microcon (amicon). Twenty microliters of purified complex was loaded onto a Superose 6 PC 3.2/30 gel filtration column (GE Healthcare precalibrated with thyroglobin [669 kDa], apoferritin [440 kDa], and γ-globulin [158 kDa]) in C-100^∗^ buffer (20 mM HEPES, pH 7.6, 10% glycerol, 100 mM KCl, 1.5 mM MgCl_2_, 0.2 mM EDTA, 0.02% NP40, 0.5 mM DTT, protease inhibitor cocktail [Roche]) fractions were collected at a flow rate of 20 μl/min and analyzed by western blotting with the indicated antibodies. For analysis of native complexes approximately 450 μl of nuclear extract was separated in a gel filtration superose 6 10/300 GL column (GE healthcare, precalibrated with Mix 2 [thyroglobulin 669 kDa, aldolase 158 kDa, ovalbumin 43 kDa and Mix 1 [dextran blue 2 mDa, ferritin 440 kDa, conalbumin 75 kDa), both from GE Healthcare] in BC300 buffer (300 mM KCl, 10% glycerol, 50 mM HEPES, pH 7.9, 0.5 mM EDTA, 0.5 mM DTT. Two hundred microliter fractions were collected, and 20% was analyzed by western blot with the indicated antibodies.

**Immunoprecipitation of Native PRC1-like Complexes**

For immunoprecipitation of native PRC1-like complexes, nuclear extracts were prepared from 6 x 10^8^ Eed4 WT or Eed4 cKO cells, and from 3.6 ml cell pellet of FSPE (Dignam et al., 1983). Five micrograms of MEL-18 (Santa Cruz), RYBP (Millipore), CBX7 (Milipore), and rabbit IgG (Santa Cruz) were crosslinked to 40 μl of Protein A magnetic beads (Invitrogen). Around 4 μg of RING1B hybridoma supernatant or 5 μg mouse IgG were crosslinked to 40 μl of Protein G magnetic beads (Invitrogen). Antibody beads were equilibrated in C-100^∗^ (20 mM HEPES, pH 7.6, 10% glycerol, 100 mM KCl, 1.5 mM MgCl_2_, 0.2 mM EDTA, 0.02% NP40, 0.5 mM DTT, protease inhibitor cocktail [Roche]), and blocked with 0.1 mg/ml insulin (Sigma), 0.2 mg/ml chicken egg albumin (Sigma), and 1% fish skin gelatin (Sigma). One hundred and fifty microliters of nuclear extract from either Eed4 WT or Eed4 cKO was diluted to 550 μl in dialysis buffer D, and 300 μl of FSPE nuclear extract diluted to 550 μl was incubated with 40 μl of antibody beads in the absence of Benzonase (Benzo) and EtBr or with either 25 U Benzonase (Novagen) and/or 2.5 μg/ml EtBr for 3 hr in no stick microcentrifuge tubes. Immunoprecipitations were washed five times for 20 min with C-100^∗^ at 4°C, and eluted from beads by incubation with SDS-loading dye for 1 hr at RT. 10% Input and 15% of RING1B, MEL-18, RYBP, CBX7, and the appropriate control coimmunoprecipitations were analyzed by SDS polyacrylamide gel electrophoresis.

**Recombinant PRC1 Complexes**

Full-length RYBP and CBX7 were tagged with HA and cloned into pDEST8 using the gateway cloning system (Invitrogen). MEL-18 and RING1B were described previously (Elderkin et al., 2007). Recombinant Baculovirus were generated using the Bac-to-Bac system (Invitrogen). vMEL18-HA, vRYBP-HA, vCBX7-HA, and RING1B-Flag-Hisx2 were coinfected in Sf9 cells for 60 hr. MEL-18/RING1B, MEL-18/RING1B/RYBP, MEL-18/RING1B/CBX7, and RYBP were purified as described previously (Elderkin et al., 2007).

Ubiquitylation assays were performed as described previously (Elderkin et al., 2007), using 300 ng E1 (Boston Biochem), 300 ng UbcH5c (affinity), 1 μg ubiquitin (Sigma), 0.0037 MBq I125-Ub (Perkin Elmer), the indicated concentration of E3, and 1.5 μg oligonucleosome. I125-ubiquitin products were visualized and quantified using Fuji FLA 3000 phosphoimager and AIDA software.

**shRNA Plasmid Construction, Lentivirus Production, mESC Infection**

Three micrograms of shRNA oligonucleotide RYBP sh2 (CCGGCCAGGAAACCTCGCATCAATTCTCGAGAATTGATGCGAGGTTTCCTGGTTTTTG), RYBP sh3 CCGGCAGCAGTGAATGATGAATCTTCTCGAGAAGATTCATCATTCACTGCTGTTTTTG (designed by Sigma) or scrambled (Sigma) were annealed in annealing buffer (100 mM NaCl, 50 mM HEPES, pH 7.9) for 4 min at 94°C and cooled overnight at RT. Annealed oligos were then ligated into the pLKO1 plasmid digested with AgeI and EcoRI. Correct plasmids were transfected into 293T cells for lentiviral production. Specific shRNA plasmids were mixed with helper plasmids, 0.4 M CaCl_2_ and 2 x HEBS (280 mM NaCl, 10 mM KCl, 1.5 mM Na_2_HPO_4_-2H_2_O, 12 mM dextrose/glucose, 50 mM HEPES, free acid, pH 7.05). The mix was added gently to cells fed with 9 ml of fresh medium and virus was collected at 48 hr and 72 hr.

*Eed^+/+^* and *Eed*^−/−^ mESCs were preplated to remove PEFs and 7.5 × 10^5^ cells were plated in a 6-well dish with 450 μl of virus and 8 μg/ml of polybrene with fresh medium. Cells were split the following day into a 90 mm dish with PEFs. Forty-eight hours after infection selection was started with 1.75 μg/ml puromycin. When colonies emerged they were individually picked and expanded for analysis.

**Immunofluorescence Analysis**

IF analysis of H2AK119u1 and H3K27me3 Xi foci in E7.5 embryos from crosses between *Eed^3354SP^* heterozygotes was carried out essentially as described previously (de Napoles et al., 2004; Silva et al., 2003). For mESCs, cells were split the previous day onto coverslips coated with PBS 1% gelatine. 36^EedTg^ and 36^Eed−/−^ mESC IFs were performed as described previously (Fang et al., 2004). Antibodies used were H2AK119u1 (05-678, Millipore) 1:10 and RYBP (ab5976, Abcam) 1:1000. Secondary antibodies used were Alexa Fluor 488 goat anti-rabbit IgG (H^+^L) (A11008, Molecular Probes) 1:400 and Alexa Fluor 568 goat anti-mouse IgG (H^+^L) (A11031, Molecular Probes) 1:400.

## Accession Numbers

ChIP sequencing data are available at GEO under accession number GSE23716. Primers used in this study are in Table S1.

## Supplemental Information

Table S1. PCR Primers, Related to Experimental Procedures

Document S1. Article plus Supplemental Information
