# Supplementary material for: RYBP-PRC1 Complexes Mediate H2A Ubiquitylation at Polycomb Target Sites Independently of PRC2 and H3K27me3
Source: Cell. 2012 Feb 17;148(4):664–78. doi: 10.1016/j.cell.2011.12.029 (PMC3281992; doi:10.1016/j.cell.2011.12.029)
Supplement: Table S1. PCR Primers, Related to Experimental Procedures [file mmc1.pdf]

Table S1. PCR Primers, Related to Experimental Procedures

ChIP primer name	Sequence (5'-3')
Oct4F	GTGAGCCGTCTTTCCACCAGG
Oct4 R	GGGTGAGAAGGCGAAGTCTGAA
Sox2F	CCATCCACCCTTATGTATCCAAG
Sox2R	CGAAGGAAGTGGGTAAACAGCAC
NanogF	CCCAGGTTTCCCAATGTGAAG
NanogR	AAAGAGTCAGACCTTGCTGCCA
mRPS14F	GACCAAGACCCCTGGACCT
mRPS14R	CCCCTTTTCTTCGAGTGCTA
EnsaF	ATGCAACATTCAGCGACTTG
EnsaR	CTAGAACACCAACCGGGAAA
B2mF	GCATCAACAGCTAGGGACTGGTGAC
B2mR	CCGAGTAGCAGCCACTGAAATGAG
Gata4F	AAGAGCGCTTGCGTCTCTA
Gata4R	TTGCTAGCCTCAGATCTACGG
Math1F	CCTTCTTTGACTGGGCAGAC
Math1R	ACTCGGAGATCGCACACC
Nkx2-2F	CAGGTTCGTGAGTGGAGCCC
Nkx2-2R	GCGCGGCCTCAGTTTGTAAC
Hoxa7F	GAGAGGTGGGCAAAGAGTGG
Hoxa7R	CCGACAACCTCATACCTATTCCTG
Msx1F	ACAGAAAGAAATAGCACAGACCATAAGA
Msx1R	TTCTACCAAGTTCCAGAGGGACTTT
Cdx2F	GGACTCCGCGAGCCAA
Cdx2R	CTCAGCCCACGGTGCTC
Pax3F	CGGACGAGTTTGGTGCGAGT
Pax3R	TGTTCCTGCCTCGTCACCAC
Sox1F	ACAAGAGGAGGCAGCGAACC
Sox1R	TCGCAGGTGGAAAGTTTCTCC
Gata1F	AGAGGAGGGAGAAGGTGAGTG
Gata1R	AGCCACCTTAGTGGTATGACG
**RT-PCR primer name**	**Sequence (5'-3')**
Gapdh F	CCCACTTGCCTCTGTATTGG
Gapdh R	CTGTGGGGAGTCCTTTTCAG
IdhF	AGAAAATGTGGAAGAGCCCTAACG
IdhR	TGCCAGCTCGATCTACCACAAAAT
HmbsF	CTGTACCTGACTGGTGGAGTCTGGAGTC
HmbsR	TGATGCCCAGGTTCTCAGCAGCT
Oct4F	CGTGGAGACTTTGCAGCCTG
Oct4 R	GCTTGGCAAACTGTTCTAGCTCCT
Sox2F	CATGTGAGGGCTGGACTGCG

NanogF	GAACTATTCTTGCTTACAAGGGTCTGC
NanogR	GCATCTTCTGCTTCCTGGCAA
Gata4F	GAGGCTCAGCCGCAGTTGCAG
Gata4R	CGGCTAAAGAAGCCTAGTCCTTGCTT
Cdx2F	TCAACCTCGCCACAACCTTCCC
Cdx2R	TGGCTCAGCCTGGGATTGCT
Pax3F	CCCAGTGAGAGGGGAGAGAGCATA
Pax3R	CTTCGAACGCAGACAGCAGC
Gata1F	GTCCTCACCATCAGATTCCACAG
Gata1R	AGTGGATACACCTGAAAGACTG
